# Multimodal epigenetic changes and altered NEUROD1 chromatin binding in the mouse hippocampus underlie FOXG1 syndrome

**DOI:** 10.1073/pnas.2122467120

**Published:** 2023-01-04

**Authors:** Ipek Akol, Annalisa Izzo, Fabian Gather, Stefanie Strack, Stefanie Heidrich, Darren Ó hAilín, Alejandro Villarreal, Christine Hacker, Tudor Rauleac, Chiara Bella, Andre Fischer, Thomas Manke, Tanja Vogel

**Affiliations:** ^a^Department Molecular Embryology, Institute for Anatomy and Cell Biology, Medical Faculty, University of Freiburg, 79104 Freiburg, Germany; ^b^Faculty of Biology, University of Freiburg, 79104 Freiburg, Germany; ^c^AUC School of Medicine, University of Central Lancashire, Preston, UK; ^d^Laboratorio de Neuropatología Molecular, Instituto de Biología Celular y Neurociencia “Prof. E. De Robertis” UBA-CONICET, Facultad de Medicina, Universidad de Buenos Aires, Buenos Aires 1121, Argentina; ^e^Max Planck Institute of Immunobiology and Epigenetics, 79108 Freiburg, Germany; ^f^Department for Systems Medicine and Epigenetics, German Center for Neurodegenerative Diseases (DZNE), Göttingen 37075, Germany; ^g^Cluster of Excellence “Multiscale Bioimaging: From Molecular Machines to Networks of Excitable Cells”, University of Göttingen, Göttingen 37075, Germany; ^h^Department of Psychiatry and Psychotherapy, University Medical Center Göttingen, Göttingen 37075, Germany; ^i^Center for Basics in NeuroModulation, Medical Faculty, University of Freiburg, Freiburg 79104, Germany

**Keywords:** Rett-syndrome, histone modification, neuronal differentiation, ATAC, axonogenesis

## Abstract

Forkhead box G1 (FOXG1) has important functions in neuronal differentiation and balances excitatory/inhibitory network activity. Thus far, molecular processes underlying FOXG1 function are largely unexplored. Here, we present a multiomics data set exploring how FOXG1 impacts neuronal maturation at the chromatin level in the mouse hippocampus. At a genome-wide level, FOXG1 i) both represses and activates transcription, ii) binds mainly to enhancer regions, iii) reconfigures the epigenetic landscape through bidirectional alteration of H3K27ac, H3K4me3, and chromatin accessibility, and iv) operates synergistically with NEUROD1. Interestingly, we could not detect a clear hierarchy of FOXG1 and NEUROD1, but instead, provide the evidence that they act in a highly cooperative manner to control neuronal maturation. Genes affected by the chromatin alterations impact synaptogenesis and axonogenesis. Inhibition of histone deacetylases partially rescues transcriptional alterations upon FOXG1 reduction. This integrated multiomics view of changes upon FOXG1 reduction reveals an unprecedented multimodality of FOXG1 functions converging on neuronal maturation. It fuels therapeutic options based on epigenetic drugs to alleviate, at least in part, neuronal dysfunction.

## Significance

Mutations in the *FOXG1* gene cause a rare neurodevelopmental disorder called “FOXG1-syndrome”. FOXG1 is a key instructor of the developing telencephalon, and patients present with various phenotypes including microcephaly, seizures, and cognitive dysfunctions. We explored the pleiotropy of molecular changes underlying neuronal abnormalities upon loss of FOXG1 and provide the multiomics data set exploring functions of mouse FOXG1 at the chromatin level. We report changes in the epigenetic landscape, impacting the accessibility of chromatin regions and activation of enhancers, upon reduced FOXG1 expression that alter the transcriptome in mouse hippocampal neurons. We identified cooperation of FOXG1 with the proneuronal transcription factor NEUROD1 and HDACs in controlling gene transcription, indicating complex and multimodal FOXG1 functions regarding neuronal maturation and function.

Forkhead box G1 (FOXG1)-syndrome is a rare, congenital neurodevelopmental disorder. Patients present with a complex phenotypic spectrum including microcephaly, seizures, severe cognitive dysfunction (1–3), and hallmarks of autism (4). Therapeutic options remain limited for patients with FOXG1-syndrome.

In the mouse, FOXG1 acts as one key determinant in central nervous system (CNS), especially forebrain development (5). In the developing ventral and dorsal telencephalon, FOXG1 affects progenitor proliferation and differentiation, and impacts corticogenesis (5–10).

Less information is available describing FOXG1 functions in the postnatal brain. In mature cerebellar neurons, FOXG1 interacts with one of two MECP2 isoforms (MECP2-e2) to prevent cell death (11). In the postnatal hippocampus, FOXG1 also prevents cell death of postnatally born dentate gyrus (DG) neurons and fosters hippocampal progenitor proliferation and differentiation (12, 13). *Foxg1*-haploinsufficiency in the mouse hippocampus results in hyperactivity, impaired habituation in open-field tests, reduced performance in contextual fear conditioning (13), and autism-like features (14). Altered levels of FOXG1 in mice impair electrophysiological properties in neurons and disbalance neuronal excitatory or inhibitory functions, stunt dendritic complexity, and reduce spine densities (14–18). Thus, features of FOXG1 alterations in mice reflect those seen in human FOXG1-syndrome patients, but FOXG1 molecular functions are yet to be fully elucidated. Many mouse studies have relied on the complete loss of both *Foxg1* alleles, and thus cannot fully recapitulate loss of a single *Foxg1* allele. We hypothesized that heterozygous animals and cell models expressing reduced levels of FOXG1 upon shRNA-mediated knockdown (KD) would advance our understanding of FOXG1-mediated molecular alterations. Importantly, delineation of the molecular mechanisms of FOXG1-syndrome was scarce, despite FOXG1 having been recognized as a key transcription factor (TF) of forebrain development for many years. In-depth analyses of chromatin-related and chromatin-independent FOXG1 functions during forebrain development are still limited (5, 7, 19–21).

Mouse mutants and human features of FOXG1-syndrome indicate that impaired FOXG1 function in the hippocampus might account for some of the phenotypes observed, rationalizing to study mouse hippocampal neurons as model system. To decipher general FOXG1 functions at the chromatin level, we reduced FOXG1 levels through shRNA-mediated KD and used a multiomics approach to unravel FOXG1’s actions at the chromatin level. We exemplarily validated our findings in a mouse model in which one allele of *Foxg1* was replaced by the cre recombinase (Foxg1^cre/+^). Our data show that FOXG1 mainly binds to enhancer regions, reconfigures the epigenetic landscape by altering chromatin accessibility, and alters H3K27ac and H3K4me3 marks at enhancers. Furthermore, FOXG1 cooperates with HDACs and NEUROD1 to regulate expression of genes that affect terminal differentiation/maturation of neurons.

## Results

### FOXG1 Regulates Gene Expression in Mouse Hippocampal Neurons In Vivo and In Vitro.

FOXG1-syndrome is associated with impaired hippocampal functions, albeit the underlying molecular mechanism is not well understood. We thus used hippocampal neurons to investigate how FOXG1 controls transcription at the chromatin level.

**Fig. 1.**
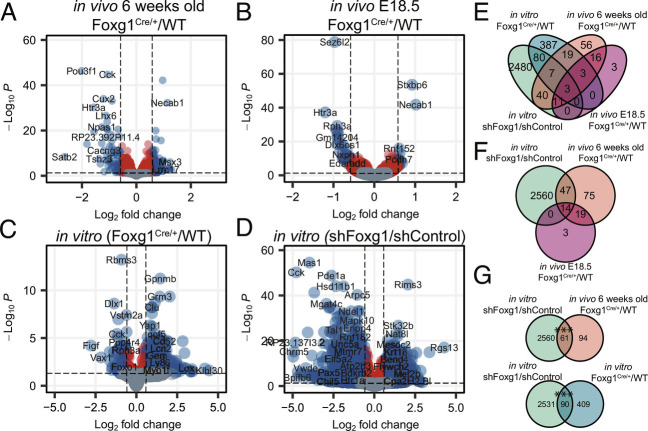
Foxg1^cre/+^ or shRNA-mediated knockdown alters the transcriptome of the mouse hippocampus during development, in vivo and in vitro. Volcano plots displaying DEGs between hippocampal samples from (*A*) 6-wk-old (n = 2), (*B*) E18.5 (n = 3) *Foxg1*^cre/+^ and *Foxg1*^+/+^ mice, and DIV11 primary hippocampal neurons from (*C*) *Foxg1*^cre/+^ and *Foxg1*^+/+^ (n = 3), (*D*) WT embryos with *Foxg1* and *Luciferase* shRNA KD (n = 4). Y-axes: adjusted *P*-value; X-axes: log_2_ fold change (log_2_FC). Gray: Insignificant adjusted *P*-values (*P *> 0.05). Red: Differential expression of less than ±log_2_(1.5). Blue: Differential expression of more than ±log_2_(1.5). Positive log_2_FC represents increase, negative log_2_FC decrease in expression upon Foxg1^cre/+^ or shRNA-mediated KD. Venn diagram of DEGs present in all four data sets (*E*), DEGs present in DIV11 shFoxg1/shLuciferase (green), 6 wk old (orange) and E18.5 *Foxg1*^cre/+^/*Foxg1*^+/+^ (pink) data sets (*F*), and pairwise comparisons of DIV11 shFoxg1/shLuciferase (green) and 6 wk old (orange) (*Top*), or DIV11 shFoxg1/shLuciferase (green) and *Foxg1*^cre/+^/*Foxg1*^+/+^ (blue) data sets (*G*). (*F* and *G*) *GeneOverlap Fisher’s exact test* ***P < 0.001.

The DG as well as CA field granule neurons expressed *Foxg1* in vivo. As shown in *SI Appendix*, Fig. S1*A* and reported by others (13, 22, 23) the *Foxg1*-heterozygous (Foxg1^cre/+^) adult hippocampus contained all fields, but was smaller compared with wild type (WT). Foxg1^cre/+^ reduced *Foxg1* transcript levels at E18.5 and in the adult (*SI Appendix*, Fig. S1*B*), but only reduced protein levels in the developing hippocampus (*SI Appendix*, Fig. S1 *C* and *D*).The expression of granule cell marker genes in the adult showed neither obvious nor significant quantitative differences between the genotypes (*SI Appendix*, Fig. S1 *E* and *F*), and the trisynaptic neuronal network of the hippocampus was similarly unchanged (*SI Appendix*, Fig. S1*G*). Regardless of a compensatory regulation of FOXG1 protein expression in vivo, RNA-seq revealed transcriptomic changes in Foxg1^cre/+^ adult hippocampus compared with WT, displaying 174 DEGs (105 increased, 69 decreased) within a threshold of log_2_ fold change (LFC) ±log_2_(1.5) and an adjusted *P*-value ≤0.05 (Fig. 1*A*). Comparing our adult transcriptome data with region-specific transcriptomes of the hippocampus (24) revealed that FOXG1 haploinsufficiency in vivo not only affected the DG (13), but also increased expression of CA3/4-field-enriched marker genes, and decreased expression of CA1-field-enriched transcripts (*SI Appendix*, Fig. S2 *A* and *B*). Thus, FOXG1 acts in a cell-type-specific manner in the adult hippocampus, and has presumably different functions/target genes during development.

To untangle molecular functions of FOXG1, we favored an earlier, i.e., less complex, developmental time point. Thus, we analyzed the transcriptomes of E18.5 Foxg1^cre/+^ hippocampal tissue (Fig. 1*B*), cultivated E18.5 Foxg1^cre/+^ primary hippocampal neurons (Fig. 1*C*), and WT primary hippocampal neurons upon FOXG1 KD at DIV (days in vitro) 11 (Fig. 1*D*) compared with their respective controls. We confirmed that both FOXG1 transcript and protein levels significantly decreased (LFC of 1.5 and 2) upon FOXG1 KD (*SI Appendix*, Fig. S1 *B*–*D*). Notably, the shRNA-mediated KD of FOXG1 in cultivated primary hippocampal neurons in vitro allowed the exploration of general and acute FOXG1 functions in a comparably homogeneous cell context, without confounding developmental effects of Foxg1^cre/+^. Foxg1^cre/+^ led to transcriptional changes in E18.5 hippocampus (36 DEGs, 11 increased, 25 decreased) and in vitro DIV11 primary neurons (504 DEGs, 448 increased, 56 decreased) (Fig. 1 *B* and *C*). As expected, FOXG1 KD compared with luciferase KD had a more pronounced effect on the transcriptome and retrieved 2626 DEGs (821 increased, 1,805 decreased) (Fig. 1*D*). Of these, increased DEGs enriched in functional terms such as *forebrain development*, whereas *neuronal differentiation, axonogenesis*, and *synaptogenesis* dominated DEGs with decreased expression (*SI Appendix*, Fig. S3 *B* and *D*).

To assess whether the KD in vitro model represented the transcriptional profile of cells with genetic reduction in *Foxg1*, we determined the number of overlapping DEGs between the different transcriptomes (Fig. 1*E*). FOXG1 KD had a higher number of shared DEGs with the in vivo adult Foxg1^cre/+^ hippocampus data set compared with the in vivo E18.5 Foxg1^cre/+^ hippocampus, indicating that the KD rather modeled a developmentally advanced stage compared with E18.5 tissue in vivo (Fig. 1*F*).

Sixty-one DEGs overlapped between FOXG1 KD and adult Foxg1^cre/+^ hippocampus, and 90 DEGs between FOXG1 KD and in vitro cultivated E18.5 Foxg1^cre/+^ primary neurons. Although these numbers were a small fraction of the total number of DEGs detected upon FOXG1 KD, the number of overlapping DEGs was significantly higher than expected for random samples (Fig. 1*G*). As anticipated, the transcriptional profiles using the differing model systems varied substantially, but we concluded that despite the differences in the four model systems of *Foxg1* reduction, the degree of overlap of the FOXG1 KD with the other models had sufficient significance to render the KD model suitable for further mechanistic analyses focusing on acute FOXG1 functions in maturing hippocampal neurons.

We characterized DEGs in the different model systems determining enriched gene ontology (GO) terms, which are indicative of phenotypic downstream effects of *Foxg1* reduction. GO-term enrichment considering all four data sets retrieved several shared functional terms, six of which overlapped between KD and both in vivo Foxg1^cre/+^ hippocampus data sets (adult, E18.5), including *synapse organization, regulation of membrane potential,* and *neurotransmitter transport* (*SI Appendix*, Fig. S3*A*). GO-term analysis of increased and decreased DEGs from the in vivo Foxg1^cre/+^ (adult) and KD data sets indicated that some functional terms were enriched in the same fractions, while others were enriched in opposite fractions (*SI Appendix*, Fig. S3*B*). *Synapse organization* was enriched in the decrease fraction in both data sets, while *extracellular matrix organization* was in the increased and decreased fractions in vivo and in vitro, respectively. These differences were also evident when both adult Foxg1^cre/+^ hippocampus and FOXG1 KD data sets were analyzed separately, to visualize functions that were ranking lower than the top 10 terms in the common analysis (*SI Appendix*, Fig. S3 *C* and *D*).

Summarizing, FOXG1 KD in DIV11 cultured hippocampal neurons and Foxg1^cre/+^ in vivo in the adult hippocampus affected similar functions, albeit not always in the same direction, which might be caused by the different FOXG1 protein levels observed. FOXG1 KD in primary hippocampal neurons provided nevertheless a robust and comparable model to further study the molecular functions affected by acutely reduced levels of FOXG1, while circumventing the potential compensatory and developmental effects present in the Foxg1^cre/+^ adult hippocampus model.

### FOXG1 Binds Chromatin in Intronic and Intergenic Regions In Vivo and In Vitro.

To correlate the transcriptional changes upon FOXG1 KD or genetic reduction in hippocampus tissue or neurons with the presence of FOXG1 at the chromatin level, we performed FOXG1 ChIP-seq in WT samples, in vivo (adult tissue) and in vitro (primary neurons).

FOXG1 reduction altered transcription in both DG and CA fields, so we used hippocampus tissue subdivided into DG or CA fields for in vivo ChIP-seq. FOXG1 bound to unique loci in both the DG and CA data sets, but also to shared chromatin regions. The pattern of the distribution of FOXG1 peaks within the genome was similar in both DG and CA fields, with a high degree of overlap of functional GO-terms of genes associated with FOXG1 peaks (*SI Appendix*, Fig. S2 *C*–*F*). As we did not concentrate on region-specific FOXG1 functions in further experiments, we merged DG and CA FOXG1 peaks for in vivo ChIP-seq data sets for subsequent analyses.

**Fig. 2.**
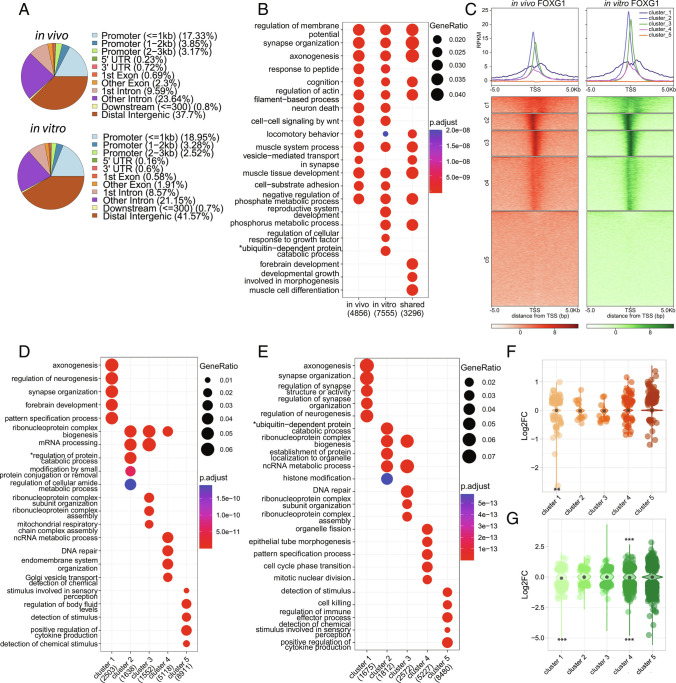
FOXG1 enriches at intergenic, intronic, and promoter regions in the hippocampus in vivo and in vitro. (*A*) Genomic distribution of FOXG1 peaks in vivo (*Top*), and in vitro (*Bottom*) and showing FOXG1 enrichment in distal intergenic (brown), intronic (pink, purple), and promoter (blue) regions. (*B*) Dotplot of differential GO-term enrichment analysis of in vivo, in vitro, and their shared FOXG1 peaks. (*C*) K-means clustering (k = 5) of FOXG1 enrichment in vivo (orange) and in vitro (green) found 5 Kb up-/downstream of TSS of protein coding genes. Data are normalized by sequencing depth and input control. The metaprofiles (*Top*) show the average reads per kilo base per million mapped reads (RPKM) of each cluster. (*D*) Dotplot of top 10 cluster-wise differentially enriched GO-terms of FOXG1 enrichment in vivo (according to clusters shown in (*A*)). *Top*-*Right* corner: Gene ratios and adjusted *P*-values. X-axis: Total number of genes per group. (*E*) Cluster-wise GO-term enrichment analysis of FOXG1 enrichment in vitro (according to clusters shown in *A*). Dotplot as in *D*. *shortened term name: “proteasome-mediated ubiquitin-dependent regulation of protein catabolic process”. (*F*) Violin plot correlating FOXG1 enrichment in k-means clusters in vivo (clusters as in *A*) and DEGs in adult Foxg1^cre/+^ hippocampus. Y-axis: log_2_FC of gene expression, X-axis: clusters. The black dot marks the median of log_2_FC of DEGs in each cluster. (*G*) Violin plot correlating FOXG1 enrichment in k-means clusters in vitro (clusters as in *A*) and DEGs upon KD of FOXG1. Representation as in *F*. (ChIP-seq n = 1 to 2). (*E* and *G*) *GeneOverlap Fisher’s exact test* *P < 0.05, **P < 0.01, ***P < 0.001. Threshold for GO-term enrichment analyses: *P *< 0.01.

For both in vivo and in vitro data, FOXG1 peaks distributed genome-wide mainly to distal intergenic and intronic regions, and a smaller fraction to promoters (Fig. 2*A*). We confirmed a significant overlap of FOXG1 peaks from in vivo and in vitro data sets, indicating high similarity of binding patterns (*SI Appendix*, Fig. S2*G*). With the transcription start sites (TSS) as reference points, we analyzed localization of FOXG1 peaks at promoters. GO-term analysis of regions where FOXG1 peaks allocated to specific genes revealed a large overlap between in vivo and in vitro data, with *neuronal differentiation* and *synaptic functions* represented within the top enriched terms (Fig. 2*B*). This was generally in line with the GO-terms based on the transcriptional changes observed in adult Foxg1^cre/+^ hippocampus and FOXG1 KD (*SI Appendix*, Fig S3 *A*–*D*).

Clustering the FOXG1 peaks near the TSS resolved further similarities between binding patterns in vivo and in vitro (Fig. 2*C*), revealing FOXG1 peaks at or near the TSS, a less discrete localization, and lack of enrichment. Cluster-wise GO-term analysis of the FOXG1 cistrome at promoter regions showed that in the first two clusters, genes with FOXG1-associated peaks affected *axono-* and *synaptogenesis* both in vivo and in vitro (Fig. 2 *D*–*F*). Genes affecting *mitochondrion organization* and *noncoding RNA biology* were represented in clusters 4 (in vivo) and 3 (in vitro). Thus, largely, cluster-wise GO-term analysis retrieved overlapping classifications in vivo and in vitro.

The distribution of DEGs upon reduced expression of FOXG1 in the five different clusters of FOXG1 peak locations for in vivo and in vitro data sets revealed DEGs in all 5 clusters, both with increasing and decreasing levels, and statistically significant enrichment of DEGs with either increased or decreased expression in specific clusters (Fig. 2 *E*–*G*). GO-term analysis pointed to FOXG1 functions in the *maturation process of neurons*. Indeed, we observed developmental dynamics in transcriptional regulation of four genes bound by FOXG1 in the adult hippocampus that affect maturation of the hippocampus (25)(*SI Appendix*, Fig. S4 *A*–*E*). Transcription of *Bdnf*, *Gria1, Gria2*, and *Syt1* increased in the adult compared with E18.5, and FOXG1 had significantly higher enrichment in *Bdnf*, *Gria1* and *Syt1* in the embryonic compared with the adult stage, indicating a repressive FOXG1 function at these loci (*SI Appendix*, Fig. S4 *C*–*E*).

We concluded that the significant overlap between in vivo and in vitro cellular resources in the binding patterns, associated gene functions, and expression changes upon FOXG1 reduction would render in vitro primary hippocampal neurons as suitable model for further experimentation in regard to acute FOXG1 functions at the chromatin level.

### Reduced Levels of FOXG1 Increase and Decrease H3K27 Acetylation.

**Fig. 3.**
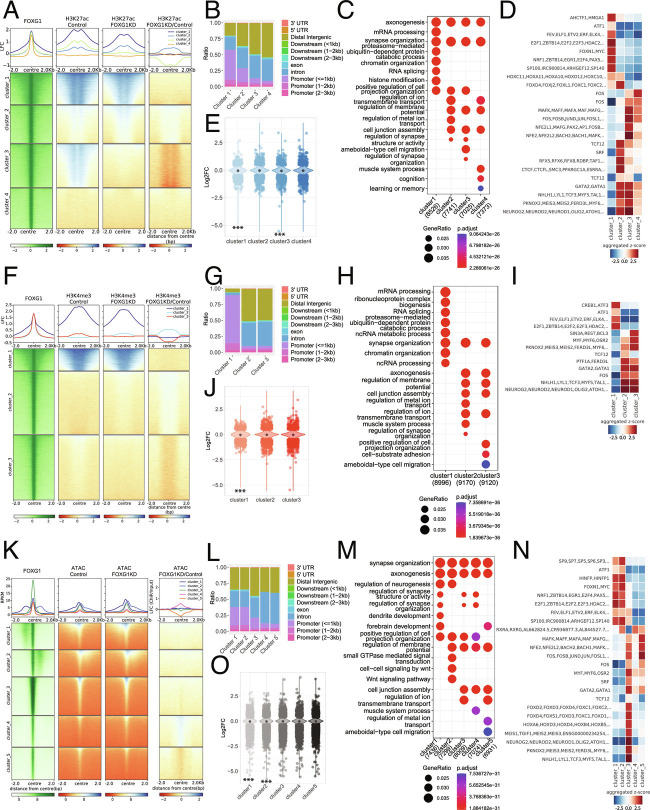
Reduced levels of FOXG1 alter the epigenetic landscape. (*A*) Heatmap of k-means clustered (k = 4) H3K27ac enrichment at 2 Kb up-/downstream of FOXG1 peak summits (*Left*, green) in control and FOXG1 KD conditions. Data are normalized by sequencing depth and input control as log_2_(ChIP/Input) for FOXG1, H3K27ac control and H3K27ac FOXG1 KD data. The difference between FOXG1 KD and control conditions was calculated from RPKM normalized bigwig files as log_2_ (FOXG1 KD/Control). The metaprofiles (*Top*) show the mean log_2_FC (LFC) of each cluster. (*B*) Genomic distribution of H3K27ac enrichment at FOXG1 peaks, according to k-means clusters from *A*, displayed as a stacked bar graph. (*C*) Top 10 differentially enriched GO-terms for the respective k-means clusters as shown in *A*. *Bottom*: Scales of gene ratios and adjusted *P*-value, X-axis: total number of genes per cluster. (*D*) TF-binding differential motif analysis according to the clusters of H3K27ac enrichment at FOXG1-binding regions as shown in *A*. Scale in Z-score. (*E*) Violin plot depicting the distribution of DEGs upon FOXG1 KD at k-means clusters of H3K27ac enrichment at FOXG1 peak as shown in *A*. Y-axis: log_2_FC of gene expression; X-axis: clusters. The black dot marks the median of log_2_FC of DEGs in each cluster. (*F*) Heatmap of k-means clustered (k = 3) H3K4me3 enrichment 2 Kb up-/downstream of FOXG1 peak summits (*Left*, green) in control and FOXG1 KD conditions. Data representation as in *A*. (*G*) Genomic distribution of clustered H3K4me3 enrichment at FOXG1 peaks. (*H*) GO-term analysis of clustered H3K4me3 enrichment at FOXG1 peaks. Representation as in *C*. (*I*) TF-binding differential motif analysis of the three k-means clusters of H3K4me3 enrichment at FOXG1-binding regions. (*J*) Violin plot depicting the distribution of DEGs upon FOXG1 KD at the three k-means clusters (according to *F*) of H3K4me3 enrichment at FOXG1 peaks. Representation as in *E*. (*K*) Heatmap of k-means clustered (k = 5) ATAC enrichment 2 Kb up-/downstream of FOXG1 peak summits (*Left*, green) in control and FOXG1 KD conditions. Data representation as in *A*. (*L*) Genomic distribution of ATAC enrichment according to the five k-means clusters shown in *K* at FOXG1 peaks. (*M*) GO-term analysis of clustered ATAC enrichment at FOXG1 peaks. Representation as in *C*. (*N*) TF-binding differential motif analysis of the five clusters of ATAC enrichment at FOXG1 binding regions. (*O*) Violin plot depicting DEGs upon FOXG1 KD at five k-means clusters of ATAC enrichment at FOXG1 peaks. Representation and statistics as in *E*. (*E*, *J*, *O*) *GeneOverlap Fisher’s exact test* *P < 0.05, **P < 0.01, ***P < 0.001. Threshold for GO-term enrichment analyses: *P*< 0.01. (n = 2 for all data sets).

FOXG1 ChIP peaks localized at putative enhancer and promoter regions, and H3K27ac associates with transcriptional activation by marking active enhancers and promoters (26). Using quantitative ChIP-seq of H3K27ac in FOXG1 KD and control (shLuciferase) in vitro primary hippocampal neurons, we addressed the potential dynamic changes in the epigenetic landscape. Clustering of H3K27ac enrichment at FOXG1 peaks revealed co-occurrence of FOXG1 with H3K27ac in three out of four k-means clusters, thus confirming presence of FOXG1 at enhancers in hippocampal neurons (Fig. 3*A*). Reduced levels of FOXG1 in primary hippocampal neurons increased and decreased H3K27ac levels. H3K27ac enrichment did not change in cluster 1, increased in cluster 2, decreased in cluster 3, and cluster 4 did not have any noticeable levels. The regions from the H3K27ac-stable cluster 1 annotated to ~60% at promoter-assigned sites, whereas the regions with increasing (cluster 2) and decreasing (cluster 3) H3K27ac levels localized predominantly to intergenic and intronic regions (Fig. 3*B*). GO-terms of annotated FOXG1/H3K27ac-peaks in the four clusters enriched for *synapse organization* and *axonogenesis* (Fig. 3*C*). Differential TF-binding-motif analysis within 200 bp flanking the peak summits in the four clusters showed that cluster 1 regions enriched for cell-cycle control TFs, including the E2F family. Cluster 2 enriched for Forkhead (Fkh) TFs, and clusters 2, 3, and 4 enriched for basic helix–loop–helix (bHLH) and GATA motifs (Fig. 3*D*). DEGs upon FOXG1 KD distributed to all four clusters, where cluster 1 was significantly enriched in genes with decreased expression, as well as cluster 3, which contained significantly more genes with decreased expression, in accordance with reduced levels of H3K27ac (Fig. 3*E*).

We also analyzed only the regions that significantly gained or lost H3K27ac upon FOXG1 KD, as differentially enriched regions provide another level of understanding into regulatory mechanisms. Regions with significantly altered H3K27ac levels also localized predominantly to introns and intergenic regions (*SI Appendix*, Fig. S5 *A* and *B*). Gain and loss of H3K27ac enriched with genes with increased and decreased expression, respectively, indicating a better correlation between impact of FOXG1 KD on H3K27ac and altered gene expression in these targets (*SI Appendix*, Fig. S5*C*), which annotated to *axonogenesis* and *neuron projection extension* functions (*SI Appendix*, Fig. S5*D*). Specific TF motifs enriched in regions gaining or losing H3K27ac upon FOXG1 reduction (*SI Appendix*, Fig. S5*E*). To assess whether genetic reduction in *Foxg1* in vivo is also accompanied by altered levels of H3K27ac, we tested two target regions with gain or loss of H3K27ac upon FOXG1 KD, using H3K27ac ChIP-qPCR in Foxg1^cre/+^ and WT adult hippocampal tissues (*SI Appendix*, Fig. S5 *F* and *G*). The same pattern of gain or loss of H3K27ac was observed in the tissue samples and upon ChIP-seq after FOXG1 KD in vitro. These results confirmed that FOXG1 impacts H3K27ac both upon genetic deletion and after shRNA mediated KD in these two target regions.

### Reduced Levels of FOXG1 Increase and Decrease H3K4 Trimethylation.

H3K4me3 associates with enhancers and active/poised promoters. To investigate whether FOXG1 controls transcription also by affecting this epigenetic modification, we generated and analyzed H3K4me3 ChIP-seq data from primary hippocampal neurons. To explore the H3K4me3 enrichment profile at FOXG1-binding sites, we used k-means (k = 3) clustering in FOXG1 KD data set compared with control (shLuciferase), and showed that two clusters displayed mild changes in H3K4me3 levels. Cluster 2 had slightly increased, and cluster 3 had slightly decreased levels of H3K4me3 (Fig. 3*F*). The majority of the H3K4me3 peaks localized in cluster 1, at promoters, whereas the regions gaining or losing H3K4me3 upon FOXG1 KD mapped with 90% to intronic or intergenic regions, supporting the impact of FOXG1 on the epigenetic landscape at enhancers (Fig. 3*G*). *Synaptogenesis* and *axonogenesis* were among enriched GO-terms annotated to dynamic H3K4me3 clusters (Fig. 3*H*). TF motifs enriched in the respective clusters contained bHLH and GATA TF motifs (Fig. 3*I*). DEGs upon FOXG1 reduction were distributed to all clusters, and significantly more genes with decreased expression were found in cluster 1, which had the strongest H3K4me3 levels (Fig. 3*J*). Focused analyses on the regions with significant gain or loss of H3K4me3 upon FOXG1 KD showed that these regions were classified mainly as promoters (*SI Appendix*, Fig. S6 *A* and *B*), DEGs with decreased expression were significantly enriched in regions with decreased H3K4me3 (*SI Appendix*, Fig. S6*C*), and *axon extension* enriched as GO-term within the genes that lost H3K4me3 alongside other terms indicative of *neuronal differentiation* (*SI Appendix*, Fig. S6*D*).

### Reduced Levels of FOXG1 Increase and Decrease Chromatin Accessibility.

Taking into account the presence of FOXG1 at enhancers, its action toward epigenetic marks, and the prediction that other TFs might be affected at these locations, we resolved to analyze alterations in chromatin accessibility upon FOXG1 KD using ATAC-seq. Indeed, FOXG1 peaks showed varying chromatin accessibility. Of the k-means clustered peaks, cluster 4 showed increased accessibility and clusters 3 and 5 decreased accessibility upon FOXG1 KD (Fig. 3*K*). As with the other epigenetic parameters, the altered clusters mainly contained intergenic and intronic regions (Fig. 3*L*). Similarly, GO-terms enriched for *axono-* and *synaptogenesis* (Fig. 3*M*), and Fkh, bHLH, and GATA TF motifs enriched in cluster 3 (mild decrease) (Fig. 3*N*). DEGs distributed again to all clusters, with clusters 1 and 2 showing significant enrichment of DEGs with decreased expression, although these clusters did not show any changes in accessibility upon FOXG1 KD, indicating other mechanisms at play at these already accessible regions (Fig. 3*O*).

The focused analyses of the regions that significantly altered accessibility upon FOXG1 KD affirmed impact at enhancer regions (*SI Appendix*, Fig. S7 *A* and *B*). Compared with H3K27ac and H3K4me3, the FOXG1 peaks distributed more sharply around the center of the peak in the regions that changed in accessibility (*SI Appendix*, Figs. S5*A*, S6*A*, and S7*A*). All clusters contained DEGs (*SI Appendix*, Fig. S7*C*) and *synaptogenesis* enriched as a GO-term within the genes that gained access, while *signal transduction* was enriched within genes that lost access (*SI Appendix*, Fig. S7*D*). bHLH and GATA TF motifs also enriched in regions gaining access upon FOXG1 reduction (*SI Appendix*, Fig. S7*E*).

Summarizing the entire epigenetic survey, FOXG1-bound chromatin localized to enhancers, and to a smaller extent to promoter regions. The epigenetic alterations studied upon FOXG1 KD affected H3K27ac and H3K4me3 levels as well as chromatin accessibility, whereby both gain and loss of marks/accessibility was observed. Affected genes classified as regulating axono- and synaptogenesis. Epigenetic changes impacted regions that can be bound by TFs, among which bHLH family members were associated in all three altered chromatin contexts. Thus, FOXG1 is a TF with pleiotropic activities at the chromatin level, one of which is alteration of the epigenetic landscape.

### HDAC Inhibition Reverts a Fraction of FOXG1 Transcriptional Changes In Vitro and In Vivo.

**Fig. 4.**
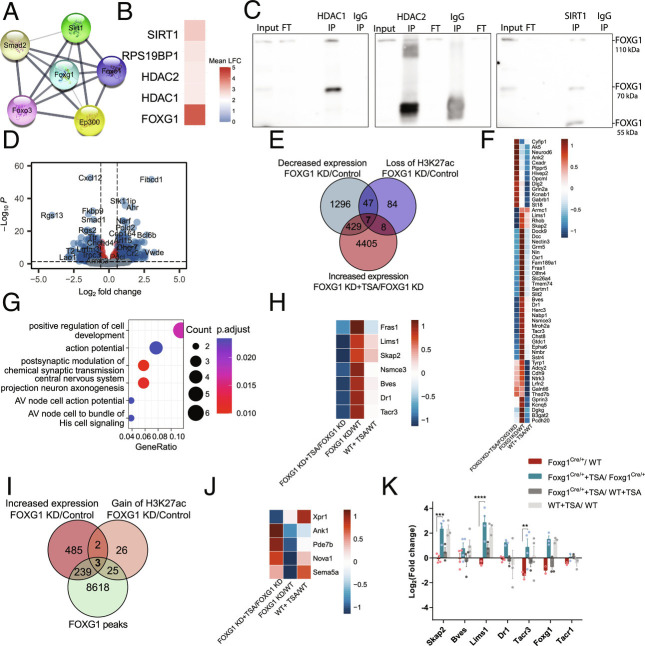
FOXG1 influences HDAC function both in vitro and in vivo. (*A*) STRING DB predictions of FOXG1-interacting HATs and HDACs. Line thickness indicates the strength of data support. (*B*) Heatmap of enriched HDACs upon FOXG1 pull-down after its overexpression in N2A cells according to (27). (*C*) Immunoblots after co-IP, demonstrating interaction between HDAC1, HDAC2, SIRT1 and FOXG1 in hippocampal tissue from adult WT animals (n = 1). (*D*) Volcano plot showing 992 DEGs of DIV11 hippocampal neurons upon TSA treatment alongside FOXG1 KD (n = 4). DEGs from FOXG1 KD+TSA/FOXG1 KD+DMSO analysis were intersected with DEGs from FOXG1 KD/Control analysis. DEGs upon TSA treatment under control conditions were removed to exclude FOXG1-independent effects of HDAC inhibition. Y-axis: adjusted *P*-value; x-axis: log_2_FC. Color code and thresholds as represented as in Fig. 1*A*. (*E*) DEGs assuming FOXG1-HDAC interaction according to repression model. Venn diagram shows the intersection of DEGs with decreased expression (green) and decreased H3K27ac (blue) upon reduced levels of FOXG1, and DEGs with increased expression upon TSA treatment (red) after FOXG1 KD. (*F*) Heatmap of 54 genes at the intersection of reduced H3K27ac and gene expression upon FOXG1 KD resulting from *E*. Scale shows log_2_FC upon respective conditions shown in the X-axis. (*G*) GO-term enrichment analysis shows the top biological processes affected in the 54 genes according to repression model. (*H*) Heatmap of 7 genes at the intersection of reduced H3K27ac and gene expression upon FOXG1 KD, and rescued upon TSA treatment. Scale as in *F*. (*I*) DEGs assuming FOXG1-HDAC interaction according to recruitment model. Venn diagram demonstrates the intersection of FOXG1 peaks (green), DEGs with increased expression (red) and gain of H3K27ac (orange) upon reduced levels of FOXG1. (*J*) Heatmap of 5 genes at the intersection of gain of H3K27ac and increased gene expression upon Foxg1 KD assuming the recruitment model resulting from *I*. Scale as in *F*. (*K*) qRTPCR validation of the repression model in vivo. Systemic TSA treatment of 6 wk old Foxg1^cre/+^ mice rescued (blue) the reduced expression of the targets *Tacr3*, *Lims1,* and *Skap2* upon FOXG1 haploinsufficiency (red). *Tacr1* is a nontarget control, showing no rescue effect upon TSA treatment. Mean ± SEM, Two-way ANOVA with Tukey’s multiple comparisons: *P < 0.05, **P < 0.01, ***P < 0.001. (n = 3 to 5).

To elucidate the underlying mechanism of how FOXG1 alters the epigenetic landscape, we focused on its impact on H3K27ac because of the predominant localization of FOXG1 at enhancers. We observed both increasing and decreasing levels of H3K27ac upon FOXG1 KD, but reasoned that because FOXG1 does not have HAT or HDAC activity itself, it might regulate access of these enzymes to the chromatin. Indeed, the publicly available network of proteins interacting with FOXG1 (STRING database) suggested weak association with the HAT EP300 and HDAC SIRT1 (Fig. 4*A*). Our own interactome study after the overexpression of FOXG1 in N2A cells (27) indicated potential association with HDAC1, HDAC2, and SIRT1 (Fig. 4*B*). Indeed, FOXG1 coimmunoprecipitated with HDAC1, HDAC2, and SIRT1 from adult hippocampal tissue (Fig. 4*C*), but not with EP300. However, FOXG1–EP300 interaction could be indirect via FOXO1 or FOXO3 (28) (Fig. 4*A*).

We hypothesized that manipulation of histone deacetylation with epigenetic drugs could be utilized in the development of therapeutic strategies for the human disease. Accordingly, we treated primary hippocampal neurons with the broad HDAC class I/II inhibitor Trichostatin A (TSA) upon FOXG1 KD and assessed transcriptional alterations using RNA-seq. We identified DEGs comparing FOXG1 KD with control (shLuciferase), intersected this DEG set with DEGs from TSA/DMSO upon FOXG1 KD, and excluded DEGs in TSA/DMSO in shLuciferase to eliminate FOXG1-independent effects of HDAC inhibition. This approach retrieved 992 DEGs that changed expression upon TSA treatment in FOXG1 KD (Fig. 4*D*).

FOXG1 could affect HDACs to influence gene transcription in two ways: preventing binding of HDACs to the chromatin (repression model, *SI Appendix*, Fig. S8*A*) or recruiting HDACs to the chromatin (recruitment model, *SI Appendix*, Fig. S8*B*). The repression model is independent of FOXG1-binding to the chromatin, and predicts that reduced levels of FOXG1 lead to reduced levels of H3K27ac (*shFoxg1/Ctrl decreased H3K27ac*: 156 loci) and concomitant transcriptional decrease (*shFoxg1/Ctrl decreased expression*: 1779 DEG). Upon HDAC inhibition with TSA, we expect transcriptional increase of genes (*shFoxg1+TSA/shFoxg1 increased expression*: 4849 DEG) regulated through the repression model. Intersection of the respective gene fractions showed that 54 genes decreased in expression alongside decreased H3K27ac levels (Fig. 4 *E* and *F*). GO-term analysis returned *synaptic functions* and *axonogenesis* (Fig. 4*G*). Seven genes of the 54 fulfilled all criteria for the repression model, i.e., increasing upon TSA inhibition alongside FOXG1 KD (Fig. 4 *E*–*H*). Among these target genes were *Lims1* and *Fras1*, which are linked to epilepsy and neurite growth, processes that are affected by FOXG1 mutation.

The recruitment model predicts that reduced levels of FOXG1 correlate with increased H3K27ac levels (*shFoxg1/Ctrl increased H3K27ac*: 56 loci) and concomitantly with increased transcription (*shFoxg1/Ctrl increased expression*: 729 DEG) at FOXG1-binding regions (*FOXG1 peaks*: 9066 loci) (Fig. 4*I*). In this case, TSA treatment would not alter transcription upon reduced FOXG1 levels, as inhibition of the HDACs does not occur bound to the chromatin. Five genes had increased expression and H3K27ac levels upon FOXG1 reduction, three of which were also bound by FOXG1 (Fig. 4 *I* and *J*). All criteria for the recruitment model were fulfilled by one gene, *Sema5a*, which is an autism-susceptibility gene with a role in axon guidance and synaptogenesis. Thus, FOXG1 affected HDAC functions at the chromatin level to a certain extent, whereby the repression model seemingly affected more loci than the recruitment model.

To test whether TSA application in vivo would rescue expression of target genes following the repression model, we systemically treated Foxg1^cre/+^ and WT 6-wk-old mice with TSA for 7 d and assessed the transcriptional alterations using qRTPCR (Fig. 4*K*). *Skap2, Lims1,* and *Tacr3* expression increased significantly after TSA treatment in Foxg1^cre/+^ hippocampus, while *Bves* and *Dr1* showed milder effects. *Tacr1,* a nontarget control, did not change in its expression after TSA treatment. Together, the data indicated that TSA increased the expression levels specific to HDAC interaction with FOXG1 in vivo as well as in vitro. Thus, HDAC inhibitor treatment might be worthy of further exploration as a potential pharmaceutical intervention to alleviate transcriptional alterations upon reduced FOXG1 expression.

### FOXG1 and NEUROD1 Act in Concert to Regulate Axono- and Synaptogenesis Genes in Hippocampal Neurons.

**Fig. 5.**
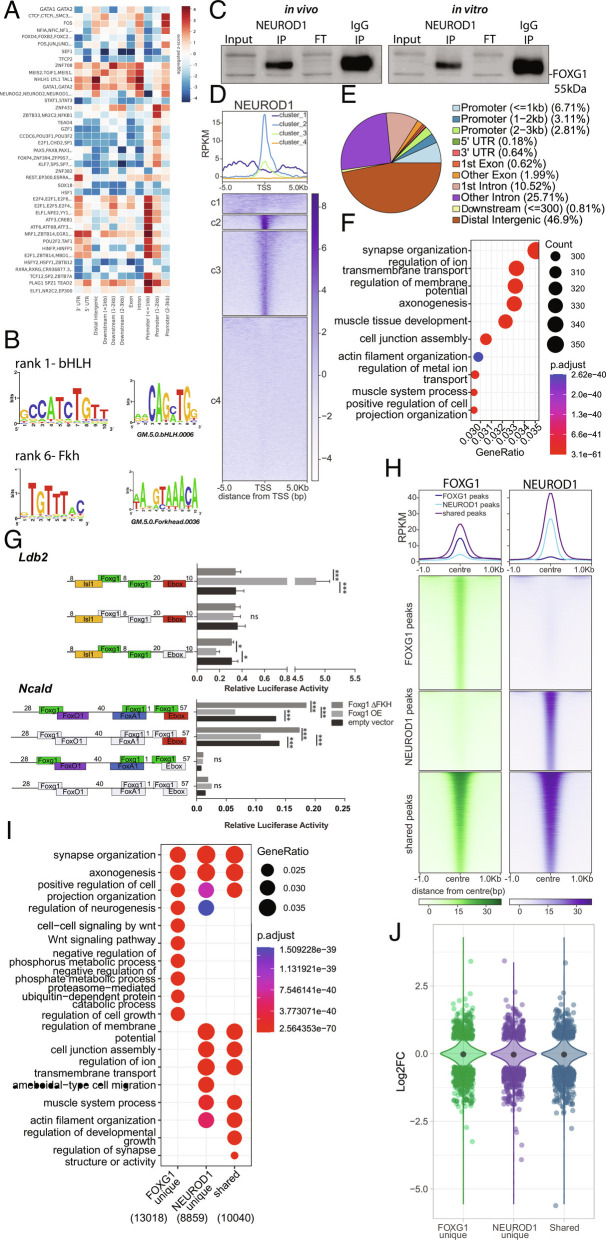
FOXG1 and NEUROD1 interact and share binding sites at genes implicated in neuronal functions. (*A*) TF-binding differential motif analysis at +/− 100 bp of FOXG1 summits stratified to genomic regions shows bHLH motif enrichment at intergenic and intronic regions. (*B*) De novo motif discovery at +/− 100 bp of FOXG1 summits include bHLH and Fkh motifs. (*C*) Representative immunoblot of FOXG1 after NEUROD1 immunoprecipitation shows that monomeric FOXG1 and NEUROD1 interact in vivo in adult hippocampus (*Left*), and in vitro in primary hippocampal neurons (*Right*) (n = 1). (*D*) K-means clustering (k = 4) of NEUROD1 enrichment in vitro at 5 Kb up-/downstream of TSS of protein coding genes. Data were normalized by sequencing depth and input control. The profiles (*Top*) show the average RPKM of each cluster. (*E*) Genomic distribution of NEUROD1 peaks in vitro shows NEUROD1 enrichment mainly in distal intergenic (brown), intronic (pink, purple) and promoter (blue) regions (n = 2). (*F*) GO-term enrichment analysis of NEUROD1 peaks. *Top* *Right*: Scales of gene ratio and adjusted *P*-value. X-axis: Total number of genes per cluster. (*G*) Luciferase reporter assays showing FOXG1 activity on regulatory regions of two target genes containing Fkh- and bHLH/E-box motifs in direct vicinity, *Ldb2* (*Top*) and *Ncald* (*Bottom*). Y-axis: Regulatory regions containing WT or modified sequences as represented in the reporter plasmids. Deleted motifs are shown in gray, while present binding motifs are shown for FOXG1 (green), FOXO1 (purple), FOXA1 (blue), bHLH/E-box motif (red), and ISL1 (yellow). X-axis: Relative luciferase activity upon co-overexpression of either full length FOXG1 (Foxg1 OE, light gray), FOXG1 with deleted Fkh-domain (Foxg1 ΔFKH, dark gray), or empty vector (control, black) with reporter plasmids. Two-way ANOVA with Tukey’s multiple comparisons *P < 0.05, **P < 0.01, ***P < 0.001, ns: nonsignificant, (n = 3). (*H*) Heatmap showing FOXG1 (green) and NEUROD1 (purple) binding sites clustered into shared and unique (FOXG1_unique, NEUROD1_unique) regions. Data normalization and metaprofiles (*Top*) as in *E*. (*I*) Dotplot shows the top 10 biological process GO-terms enriched in unique and shared clusters of FOXG1 and NEUROD1 peaks. Threshold for GO-term enrichment analyses: *P* < 0.01. (*J*) Violin plot demonstrates the distribution of DEGs upon reduced levels of FOXG1 at unique and shared clusters of FOXG1 and NEUROD1 binding sites according to *I*. Y-axis: log_2_FC of gene expression, x-axis: clusters. The black dot marks the median of log_2_FC of DEGs in each cluster.

Our exploration of the epigenetic alterations following FOXG1 reduction revealed that other TFs acted synergistically or antagonistically with FOXG1, specifically the bHLH-family proteins. Considering the reported pioneering functions of some Fkh- and bHLH-domain proteins and thus their potential to alter the epigenetic landscape (29–31), we explored whether FOXG1 acted together with bHLH TFs. We first determined the enrichment of TF-binding motifs at stratified FOXG1 peaks (Fig. 5*A*). bHLH motifs, including those of NEUROD1, NEUROD2, and NEUROG2, mainly cooccurred at FOXG1 peaks in intergenic regions and introns. This occurred with greater specificity compared with other TFs, which also enriched in other regions (Fig. 5*A*). De novo motif analysis ranked bHLH motifs at a higher significance level than Fkh motifs (Fig. 5*B*). The observation of a potential crosstalk of FOXG1 with bHLH TFs prompted us to explore this in more detail with a focus on FOXG1 and NEUROD1. We decided on NEUROD1 as its binding motif significantly enriched at FOXG1 peaks (Fig. 5 *A* and *B*), it is a proneuronal bHLH TF expressed in mature hippocampal neurons, with essential roles in neuronal development and function (32, 33) similar to FOXG1, and it has been associated with pioneering activity (29). FOXG1 and NEUROD1 coimmunoprecipitated, and they might therefore act in a concerted manner at the chromatin level (Fig. 5*C*).

We confirmed FOXG1/NEUROD1 co-occurrence on the chromatin level by determining the cistrome of NEUROD1 in primary hippocampal neurons. The NEUROD1 ChIP-seq profile, centered around the TSS, showed enrichment of NEUROD1 peaks at these sites but also up- and downstream (Fig. 5*D*), while the majority of NEUROD1 peaks localized at intergenic and intronic regions (Fig. 5*E*). Similar to what we observed for FOXG1 peak distribution, GO-terms *axono-* and *synaptogenesis* enriched in NEUROD1 peak loci (Fig. 5*F*).

We tested FOXG1/bHLH co-activity at exemplary regulatory regions of two DEGs with proximal Fkh and bHLH/E-box-binding motifs. We selected two targets, *Ldb2* and *Ncald*, that fulfilled the following specific criteria (*SI Appendix*, Fig. S9 *A*–*G*): DEGs common in in vivo and in vitro transcriptomes; DEGs with shared/close proximity of FOXG1 and NEUROD1 ChIP peaks in the promoter or gene body (exclusion of intergenic peaks); DEGs linked to reported neuronal function; bHLH and Fkh consensus motifs near or at the ChIP-peak sequence; DEGs linked to neuronal subtype specification; and DEGs with either increased (*Ldb2*) or decreased (*Ncald*) expression upon FOXG1 KD. For our luciferase assay, we used N2A cells that expressed low levels of FOXG1 and NEUROD1, alongside other Fkh or bHLH proteins (*SI Appendix*, Fig. S10 *A* and *B*), and overexpressed either FOXG1 (*SI Appendix*, Fig. S10*C*) or FOXG1 bereft of its Fkh-domain in the presence of luciferase reporter constructs. FOXG1 activation of *Ldb2* transcription was abolished upon deletion of its Fkh-domain, and was dependent on the FOXG1-binding motifs in the reporter construct. Presence of the bHLH/E-box binding sequence seemingly prevented a repressive FOXG1 function. FOXG1 repression of *Ncald* transcription was also dependent on the presence of its Fkh-domain, and the deletion of the Fkh-domain increased reporter transcription. Deletion of the binding sites for FOXG1 in the regulatory region still resulted in the same response of the reporter transcription as observed for the unmodified construct. The deletion of the bHLH/E-box-binding site silenced the regulatory region, independent of the presence or absence of Fkh motif (Fig. 5*G*).

We concluded that at these two regulatory regions, FOXG1 modulated the bHLH-mediated transcriptional regulation and that the transcriptional responses of FOXG1 in concert with bHLH appeared to be context-dependent and pleiotropic.

As our data strongly suggested cooperation between FOXG1 and NEUROD1 in transcriptional control, we compared FOXG1 and NEUROD1 ChIP-seq profiles, which returned clustered genes bound by both (shared fraction), and also returned genes that only enriched binding of one of the two TFs (FOXG1, NEUROD1 fraction) (Fig. 5*H*). Functional GO-terms that enriched in all clusters were *synapse organization* and *axonogenesis* (Fig. 5*I*). For all three clusters, including NEUROD1 unique peaks, we observed DEGs upon FOXG1 reduction, with both increased and decreased expression (Fig. 5*J*). This finding consolidated that FOXG1 and NEUROD1 act in concert, in chromatin-dependent and -independent fashions, the latter of which is displayed by altered expression of NEUROD1 uniquely bound genes upon FOXG1 KD. We clustered the observed epigenetic changes upon reduced expression of FOXG1 to the regions that were shared or enriched for either FOXG1 or NEUROD1 (*SI Appendix*, Fig. S11*A*). Epigenetic alterations were not found to be specifically enriched in any of the clusters, yet FOXG1 unique and shared clusters showed mild changes in accessibility and H3K27ac levels. For example, in the shared cluster, 1,277 regions increased and 1,695 decreased in all epigenetic markers (*SI Appendix*, Fig. S11*B*), alongside regions that had only H3K27ac, H3K4me3, or chromatin accessibility alterations, or a combination of these. We filtered and subclustered the FOXG1-NEUROD1 shared cluster that overlapped with dynamic epigenetic landscape clusters from Fig. 3 and showed that the epigenetic landscape was affected in various aspects in loci bound by both FOXG1 and NEUROD1 (*SI Appendix*, Fig. S11*C*).

Thus, alteration of the epigenetic landscape at regions bound by both TFs was moderate, indicating another mechanism by which these two key instructors affect transcriptional programs important for axono- and synaptogenesis.

### FOXG1 and NEUROD1 Act in a Concerted Manner, Both at the Chromatin Level and Prior to Chromatin Binding.

**Fig. 6.**
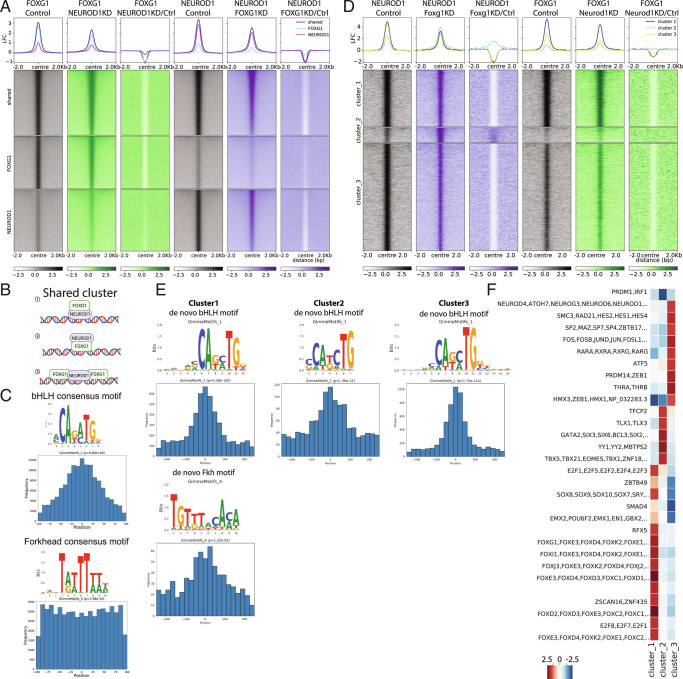
FOXG1 and NEUROD1 act in concert rather than up- or downstream from each other. (*A*) Heatmap of FOXG1 (green) and NEUROD1 (purple) enrichment clustered into unique and shared regions under control (gray), NEUROD1 KD, and FOXG1 KD conditions. Data are normalized by sequencing depth and input control as log_2_(ChIP/Input). The difference between FOXG1 KD-Control and NEUROD1 KD-Control conditions were calculated from RPKM normalized bigwig files as log_2_(TF KD/Control). The metaprofiles (*Top*) show the average log_2_FC (LFC) of each cluster. (*B*) Scheme of binding modes that classify for categorization as shared binding sites. 1: NEUROD1 binds to its bHLH/E-box motif at the chromatin and indirectly brings in FOXG1. 2: FOXG1 binds to its Fkh motif at the chromatin and indirectly brings in NEUROD1. 3: Binding sites of NEUROD1 and FOXG1 co-occur near a respective peak center (example depicts NEUROD1 as peak center). (*C*) Positional preference plots and motif logos of bHLH (*Top*) and Fkh (*Bottom*) motifs at FOXG1/NEUROD1 shared regions retrieved from de novo motif analysis. (*D*) Heatmap of k-means clustered (k = 3) NEUROD1 (purple) and FOXG1 (green) enrichment 2 Kb up-/downstream of differential NEUROD1 binding sites retrieved from *DiffBind* analysis between FOXG1 KD and control conditions. Data representation as in *A*. (*E*) Positional preference plots and motif logos of bHLH (*Top*) and Fkh (*Bottom*) motifs at sites with significant alteration of NEUROD1 binding upon FOXG1 KD, according to the three clusters from *D*, retrieved from de novo motif analysis. (*F*) Heatmap showing differential TF-binding motif analysis clustered at differential NEUROD1 binding sites as shown in *D*. (FOXG1 ChIP-seq: NEUROD1 KD n = 2, Control n = 1; NEUROD1 ChIP-seq: FOXG1 KD n = 2, Control n = 2).

To analyze the nature of the FOXG1/NEUROD1 crosstalk in more detail, we knocked down either FOXG1 or NEUROD1 in primary hippocampal neurons and assessed the binding profile of the other respective TF using ChIP-seq. We clustered the peak regions into shared fraction and regions that enriched predominantly for either FOXG1 or NEUROD1 (Fig. 6*A*). Within the shared regions, FOXG1 binding reduced upon NEUROD1 KD, and vice versa, excluding a clear hierarchy in this crosstalk.

Shared binding regions could reflect binding through one of the respective factors and indirect presence at the site of the other, or usage of adjacent binding sites (Fig. 6*B*). To resolve these binding paradigms, we determined the distribution of the respective other binding motif near either FOXG1 or NEUROD1 peaks within the shared regions. The majority of NEUROD1 motifs distributed directly in the center of the FOXG1 peaks (Fig. 6*C*). Thus, NEUROD1 most likely binds directly to chromatin with FOXG1 brought alongside, and therefore FOXG1 is indirectly recruited to chromatin at the majority of these sites. In contrast, FOXG1 motifs at NEUROD1 peaks were distributed broadly around and flanking the center of NEUROD1 peaks (Fig. 6*C*). This suggests that while FOXG1 could recruit NEUROD1, FOXG1 could also be in a cooperative binding, flanking NEUROD1 at these peaks. We concluded that rather than having a clear hierarchy within the crosstalk of both TFs, they act in a pleiotropic and cooperative manner.

Surprisingly, FOXG1 KD decreased NEUROD1 presence at NEUROD1 sites with little enrichment for FOXG1. Similarly, NEUROD1 KD decreased FOXG1 presence at FOXG1 sites with little enrichment for NEUROD1. We concluded that either factor might be also needed before or during recruitment, but not exclusively for stable binding.

We next analyzed the changes of NEUROD1 and FOXG1 binding upon the respective KDs, with a focus on regions that showed differential binding of NEUROD1 upon FOXG1 KD. Regions presenting differential binding of NEUROD1 clustered into three main profiles: Cluster 1 (strong enrichment for NEUROD1 and FOXG1), cluster 2 (moderate enrichment for NEUROD1, lower enrichment of FOXG1), and cluster 3 (strong enrichment for NEUROD1, moderate enrichment of FOXG1) (Fig. 6*D*). Upon FOXG1 KD, binding of NEUROD1 within clusters 1 and 3 reduced. Interestingly, cluster 2 had increased binding of NEUROD1 after FOXG1 KD. NEUROD1 KD also altered FOXG1 presence at loci within clusters 1 and 3, but not in cluster 2. We concluded that binding of NEUROD1 and FOXG1 at clusters 1 and 3 is cooperative, either at one binding site (cluster 3) or at adjacent sites (cluster 1), as supported by the distribution of the binding motifs in regard to the peak center (Fig. 6*E*). In contrast, cluster 2 targets are bound mainly by NEUROD1, and at these sites, FOXG1 presence interferes with NEUROD1 binding. Our differential TF-binding motif analysis of stratified clusters also supported this interpretation, as cluster 2 enriched for example for GATA and TBX TF, but neither for Fkh nor bHLH/E-box motifs (Fig. 6*F*). Thus, we speculate that NEUROD1 can be recruited to chromatin by TFs other than those of the Fkh family, but that FOXG1 is a competing NEUROD1 binding partner, impacting the cluster 2 genomic regions.

To validate FOXG1/NEUROD1 cooperation in vivo in Foxg1^cre/+^, we selected four different regions for FOXG1/NEUROD1 ChIP-qPCR. We selected the clustered regions that were differentially bound by NEUROD1 upon FOXG1 KD (Fig. 6*D*), shortlisted regions that showed the highest initial enrichment of FOXG1/NEUROD1 (depending on the cluster) and highest difference in FOXG1/NEUROD1 enrichment between conditions, and for which primer efficiency and quality allowed ChIP-qPCR analyses (*SI Appendix*, Fig. S12 *A* and *B*). Of note, FOXG1 protein levels were globally unchanged upon the genetic reduction in the adult (*SI Appendix*, Fig. S1 *C* and *D*); thus we first assessed presence of FOXG1 protein at the selected regions in the Foxg1^cre/+^ hippocampus. For clusters 1 and 2, FOXG1 levels increased at the selected sites, having the opposite appearance compared with the FOXG1 KD model (*SI Appendix*, Fig. S12*C*). The cluster 3 region was bound less in the condition of genetic deletion of one *Foxg1* allele. Enrichment of NEUROD1, on the other hand, strongly increased at the cluster 1 region. This was opposite to the KD approach, and correlated well with the increased FOXG1 presence at the site, which contained both Fkh and bHLH binding sites. The two cluster 2 regions were bound by increased amounts of NEUROD1 upon FOXG1 KD, and according to increased presence of FOXG1 at the sites in vivo, NEUROD1 levels were decreased in the Foxg1^cre/+^ hippocampus. Cluster 3 region, which did not contain enrichment of Fkh motifs, had less FOXG1 bound in the Foxg1^cre/+^ hippocampus, and in accordance with the observation of decreased NEUROD1 presence upon FOXG1 KD, we precipitated less NEUROD1 from the in vivo sample. Taken together, the validation suggests that FOXG1/NEUROD1 cooperation is of functional importance in vitro and in vivo.

## Discussion

In this study, we reveal diverse and pleiotropic functions of FOXG1 at the chromatin level in maturing neurons. Despite FOXG1 being recognized as a key TF for telencephalic development and neuronal function, insights into the mechanism underlying transcriptional regulation are sparse. Here, we show that i) FOXG1 acts both as repressor and activator, ii) localizes predominantly to enhancer regions, iii) alters the epigenetic landscape, iv) directly affects HDAC functions, and v) acts in concert with NEUROD1 to instruct transcriptional programs necessary for axono- and synaptogenesis.

FOXG1 is generally considered to be a TF with a repressive function (5, 34). However, recent data support pleiotropic and context-dependent functions, i.e., non-nuclear functions of FOXG1 encompassing posttranscriptional regulation (27) and functions in mitochondria (21). Furthermore, different signaling pathways control nuclear or cytoplasmic localization of FOXG1 (34). Chromatin-related functions of FOXG1 are only partly understood, but also seem to be diverse in their nature. For example, FOXG1 exerts transcriptional regulation by hampering FOXO/SMAD transcriptional activators to bind to *Myc*-target regions (35). The repressor ZBTB18 is cooperatively recruited with FOXG1 to genes affecting neuronal migration and axonal projections (5). We here report high-throughput data that extend current views of the multiple mechanisms used by FOXG1 to control gene expression, both as a repressor and an activator. In the hippocampus model system, we show that FOXG1 impacts regulatory genomic regions – mostly enhancers but also promoters – by direct binding and by altering the epigenetic landscape. Reduced FOXG1 levels correlated with both increased and decreased H3K27ac, H3K4me3, and/or chromatin accessibility, highlighting the diverse context-dependent molecular functions of FOXG1. We identified two means of action for FOXG1’s impact on H3K27ac: FOXG1 represses the recruitment of HDACs to chromatin (chromatin-independent function) or FOXG1 can repress HDAC function at the chromatin (chromatin-dependent function). Among FOXG1 target genes regulated via HDACs were genes associated with epilepsy, behavioral abnormalities, autism, intellectual disability, and impaired synaptogenesis (36–39). These features are also observed in FOXG1-syndrome patients.

In accordance with other studies reporting on FOXG1 cistrome in the cerebral cortex (5, 7), FOXG1 peaks localized at genes that influence maturation and function of neurons in the hippocampus. Axono- and synaptogenesis are functional terms that were enriched in several of our data sets, indicating that FOXG1 influences these processes both by direct presence, by associated epigenetic alterations, and in concert with NEUROD1. Other studies have provided experimental evidence that FOXG1 is important for axonogenesis, synaptogenesis, and other features of neuronal differentiation in the hippocampus (17), the retina (40), and the cerebral cortex (5), but lacking the mechanistic layer that our study provides.

Our data indicate that FOXG1 might have different affinities to chromatin, since regions that are moderately bound by FOXG1 are affected more from FOXG1 reduction compared with strongly bound regions. A balanced expression of FOXG1 influences proper functioning of the CNS in humans or animal (3, 14), and maintaining a critical amount of FOXG1 present at the chromatin seems important for proper neuronal function. Differences in the presence of FOXG1 at respective loci, i.e., loci with less FOXG1 bound, might confer particular vulnerability to reduction in FOXG1 levels. Of note, other Fkh TFs bind DNA as monomers and dimers (41). Our data lack the necessary resolution to resolve action as monomers or dimers, but this is an attractive model to explain one aspect of the pleiotropic effects of FOXG1 at the chromatin level.

Of particular note is our observation that the FOXG1 presence at the chromatin has a strong correlation with co-occurrence of NEUROD1, a bHLH TF necessary for neuronal differentiation (42, 43). Interestingly, both FOXG1 and NEUROD1 influenced the presence of one another at a variety of binding sites in hippocampal neurons.

NEUROD1 can act as a pioneer factor in mouse embryonic stem cells undergoing neuronal differentiation (29), where H3K27ac levels increased concomitantly with NEUROD1 activation, and H3K27me3 levels decreased at selected loci, relieving heterochromatic repression. In hippocampal neurons, we observed a concomitant reduction and increase of binding events for NEUROD1 upon reduced expression of FOXG1 In 3,150 regions that lost NEUROD1 binding upon FOXG1 KD, only 32 showed both significantly lower H3K27ac levels and differential gene expression (*SI Appendix*, Fig. S13*A*). On the other hand, in 451 regions that gained NEUROD1, only 1 region had significantly higher H3K27ac levels coinciding with changed gene expression (*SI Appendix*, Fig. S13*B*). This observation weakens support for the argument that NEUROD1 acts as a pioneer that alters H3K27ac levels in mature hippocampal neurons upon FOXG1 KD. Because of the reported pioneering potential of NEUROD1, one could hypothesize that NEUROD1 acts upstream of FOXG1, regulating its access to the chromatin. However, our data do not support a clear hierarchy of the two factors in hippocampal neurons; they rather act in concert, and regulate access to each other. Further, FOXG1 interferes with NEUROD1 binding to chromatin, similar to what has been observed for chromatin accessibility of the SMAD/FOXO TF complexes (35). Thus, FOXG1 and NEUROD1 act together in a chromatin-dependent and -independent manner.

## Conclusion

Our data highlight that the multiple modalities of FOXG1 functions in different cellular compartments or occurring at posttranscriptional and transcriptional levels extend to the chromatin level. Here, FOXG1 acts through different epigenetic mechanisms, as well as in concert with other TFs, including NEUROD1. Given that we identified a larger set of TFs enriched at FOXG1-bound peaks, including other members of the Fkh and bHLH families, the data presented unearths a small part of the wider epigenetic picture, and further studies will add to the complex pattern of different FOXG1 actions. Concerning therapeutic options, direct interference with TFs acting in concert with or antagonized by FOXG1 would prove challenging. Considering the epigenetic changes upon FOXG1 KD in light of increasing inclusion of epigenetic drugs in clinical trials, we here highlight an attractive avenue for treatments of FOXG1-syndrome by epigenetic drugs.

## Material and Methods

Detailed information on material and methods used in this study are found in the *SI Appendix*.

### Mice.

All mouse experiments were approved by the animal welfare committees of the respective authorities. *Foxg1*^cre/+^ mice were maintained in a C57Bl/6 background. As recent studies did not indicate a sex difference in heterozygote *Foxg1* mouse models (5, 13, 22, 44), this study only used male adult mice or mice from E18.5 developmental stage without discrimination between sexes.

### Viral Transduction and Selection of Primary Neurons.

Lentiviral particles were prepared using plko.1-CMV.Puro-tGFP-shNeurod1, plko.1-CMV.Puro-tGFP-shFoxg1, or plko.1-CMV.Puro-tGFP-shLuciferase (GenScript) plasmids, according to the protocol described previously (45).

### NGS Library Preparation and Sequencing.

RNA-, ChIP-, RELACS-ChIP, and ATAC-seq library preparation and sequencing were done using standard protocols as described in detail in the *SI Appendix*.

### Bioinformatics, Data Repository and Analyses of Public Databases.

The “Differential Search” tool of the Allen Brain Atlas (46) was used to define field specific gene expression. The sequencing data from RNA-, ATAC-, RELACS-, and wild-type ChIP-seq were processed with *snakePipes* (v. 1.1.1) (47). The sequencing data from FOXG1 and NEUROD1 ChIP-seq after NEUROD1 or FOXG1 KD were analyzed on the public server at usegalaxy.eu (48). Summary of quality control of all datasets is available at https://github.com/Vogel-lab/Integrative-multi-omics-analyses-of-FOXG1-functions.

Hipposeq (24) was accessed at http://hipposeq.janelia.org/ for choosing the dorsal-ventral survey of hippocampal principal cells.

STRING database (49) was used to explore known and predicted protein–protein interactions of FOXG1 in *Mus musculus*.

## Ethics Approval and Consent to Participate.

Not applicable.

## Consent for Publication.

Not applicable.

## Supplementary Material

Appendix 01 (PDF)

## Data Availability

The raw sequencing files and primary processed files were deposited to the NCBI Gene Expression Omnibus (GEO) with accession number GSE189119. All other data types and codes recreating the analyses from the data files can be found at https://github.com/Vogel-lab/Integrative-multiomics-analyses-of-FOXG1-functions as R markdown files, Python scripts, and Galaxy workflows. Previously published data were used for this work (GSE106802).
