# Supplementary material for: Multimodal epigenetic changes and altered NEUROD1 chromatin binding in the mouse hippocampus underlie FOXG1 syndrome
Source: Proc Natl Acad Sci U S A. 2023 Jan 4;120(2):e2122467120. doi: 10.1073/pnas.2122467120 (PMC9926245; doi:10.1073/pnas.2122467120)
Supplement: Supplementary file 1 — Appendix 01 (PDF) [file pnas.2122467120.sapp.pdf]

PNAS

Supporting Information for

Multimodal epigenetic changes and altered NEUROD1 chromatin binding in the mouse hippocampus underlie FOXG1 syndrome

Ipek Akol[a,b], Annalisa Izzo[a], Fabian Gather[a], Stefanie Strack[a], Stefanie Heidrich[a], Darren Ó hAilín[a,c], Alejandro Villarreal[a,d], Christine Hacker[a], Tudor Rauleac[a], Chiara Bella[e], Andre Fischer[f,g,h], Thomas Manke[e], and Tanja Vogel[a,i,1]

Corresponding author: Tanja Vogel
Email: tanja.vogel@anat.uni-freiburg.de

This PDF file includes:

Supplementary text for Material and Methods
Figures and legends S1 to S13
SI References

SI Appendix – Material and Methods

Mouse hippocampus isolation and primary neuronal culture

Hippocampi of E18.5 C57Bl/6 embryos (Charles River) were dissected and collected in 10 ml Hanks' Balanced Salt Solution (HBSS, Fisher Scientific) and dissociated in 0.25% Trypsin/EDTA (Fisher Scientific) solution at 37°C for 10 min. Dissociation was stopped by adding 10% fetal bovine serum (FBS, Fisher Scientific). Cells were collected by centrifugation and cultured in Neurobasal (NB)-complete medium, which consists of neurobasal medium (Fisher Scientific) supplemented with B27 (Fisher Scientific), L-glutamine (0.5 mM, Fisher Scientific), penicillin-streptomycin-neomycin (1X PSN, Fisher Scientific), apo-transferrin (5 µg/ml, Sigma), superoxide-dismutase (0.8 µg/ml, Sigma) and glutathione (1 µg/ml, Sigma). Cells were seeded on poly-L-ornithine (0.1 mg/ml, Sigma) and laminin (1 µg/ml, Sigma) coated 24-well plates (Corning).

Viral transduction and selection of primary neurons

Lentiviral particles were prepared using plko.1-CMV.Puro-tGFP-shNeurod1 (Genscript), plko.1-CMV.Puro-tGFP-shFoxg1 (Genscript), or plko.1-CMV.Puro-tGFP-shluciferase (Sigma MISSION) plasmids, according to the protocol described previously (1, 2). Cells were transduced with lentiviral particles on day-in-vitro (DIV) 1. At DIV4, transduced cells were selected using 0.3 µg/ml puromycin (P9620, Sigma) and cell proliferation was inhibited by addition of 2 µM Arabinocytosine (AraC, C1768, Sigma), while performing a half-volume medium change. Medium was changed again at DIV7 and DIV9 including 0.3 µg/ml puromycin and 2 µM AraC. Cells were used for either protein or RNA isolation.

shRNA sequences:

shNeurod1	CCGGGCTCAGCATCAATGGCAACTTCTCGAGAAGTTGCCATTGATGCTGAGCTTTTTG
shFoxg1	CCGGCCTGACGCTCAATGGCATCTACTCGAGTAGATGCCATTGAGCGTCAGGTTTTTG
shluciferase (Control)	CCGGCGCTGAGTACTTCGAAATGTCCTCGAGGACATTTCGAAGTACTCAGCGTTTTT

Trichostatin A (TSA) treatment of primary hippocampal neurons

3.31 mM stock TSA solution (Sigma, #T8552) was prepared in DMSO (Sigma, #02438). Primary hippocampal neurons transduced with shFoxg1 or shluciferase were treated with 0.5 µM TSA or DMSO on DIV10. TSA and DMSO dilution to the final concentration was done using neurobasal medium.

Systemic TSA injection of Foxg1$^{+/+}$/Foxg1$^{cre/+}$ mice

0.006 mg/ml TSA solution (Sigma, #T8552) was prepared in 10% DMSO (Sigma, #02438) and 90% (5% β-Cyclodextrin, Sigma) in 0.9% NaCl (Braun). 5 week old Foxg1$^{+/+}$ and Foxg1$^{cre/+}$ mice were administered (intraperitoneal injection) with 0.6 mg/kg/day TSA daily for 7 days. Mice were sacrificed at 6 weeks and the hippocampal tissue was collected and snap frozen and kept at -80 ˚C until further processing.

RNA isolation from tissue and primary hippocampal neurons

Hippocampi were frozen in liquid nitrogen immediately after dissection, and stored at -80°C until RNA extraction. Total RNA was isolated using RNeasy RNA isolation kit (Qiagen)

according to the instructions of the manufacturer. An on-column DNAse digestion was routinely performed. Isolated RNA was kept at -80°C until following qRTPCR experiments.

Cells were harvested at DIV11 in buffer RLT (RNeasy RNA isolation kit, Qiagen) for total RNA extraction according to manufacturer's instructions. Total RNA was kept at -80°C until subsequent reverse transcription.

Reverse transcription and qRTPCR

1 µg of total RNA was reverse transcribed using RevertAid MMuLV reverse transcriptase kit (Fermentas, Thermo Scientific). qRTPCR analysis was performed on a CFX-Connect Real-Time PCR detection system (Bio-Rad) using GoTaq qPCR Master Mix (Promega). Primers used had an efficiency level between 85% and 110%. qRTPCR results were analyzed using the ΔΔCt method with GAPDH as internal standard. GraphPad Prism software was used for plotting the bar graphs and for statistical analyses. Values in bar graphs are expressed as average ± SEM.

The primers for the qRTPCR target regions are listed in the following table:

**Target**	**Forward primer**	**Reverse primer**
Foxg1	AATGACTTCGCAGACCAGCA	CCGGACAGTCCTGTCGTAAA
Skap2	CCGCTATTGCGAGTCTAGGT	TCCTTTCAGTGTGTCTGCCA
Dr1	AGGCCAGTTCTCGTTTGGAA	TGGGCCAATTCTGCTTGTTG
Bves	ACGTTTGCTTTGCAGTTGGT	TGGCCCAGACCACATAAAGG
Lims1	GCGGCCGGAATGACCA	CACTGAGCACATACGAAGCAC
Tacr3	CCGTACCCTTTGCTATGTGC	GGTGTAGGTGACACCCATGA
Tacr1	CTGCTGGTGATTGGCTATGC	CGAAGGTACACACAACCACG
Gapdh	CGGCCGCATCTTCTTGTG	TGACCAGGCGCCCAATAC

ChIP-qPCR

ChIP-qPCR was performed with hippocampal tissue of E18.5 and adult mice hippocampi (6 weeks old) either wildtype (WT) or Foxg1$^{cre/+}$ snap frozen at -80°C. For one ChIP, around 10 µg of chromatin was used. Tissue was thawed on ice, resuspended in 1 ml of PBS with proteinase inhibitors (Thermo Scientific) and dounced with 11 strokes using a small glass douncer homogenizer. Cell suspension was centrifuged at 2000 g for 5 min at 4°C and cell pellets were fixed with 1.5 ml of 1.5 mM EGS (ethylene glycol bis(succinimidyl succinate), Thermo Scientific, #21565) in PBS for 30 min followed by 1 % formaldehyde in PBS for 15 min. After stopping the reaction with glycine (125mM) and washing with PBS, the cell pellets were resuspended in ChIP Buffer (Active Motif #53040) and sonicated using a Bioruptor (Diagenode) with 60 cycles at 30 sec ON/OFF. Chromatin was cleared of debris by centrifugation at maximum speed at 4°C for 5 min. Chromatin immunoprecipitation was performed as described in the protocol of the ChIP-IT High Sensitivity kit (Active Motif, #53040). Antibodies used for the immunoprecipitation were rabbit anti-FOXG1 (Active Motif, #61211), anti-NeuroD1 (Abcam, #109224), and rabbit anti-H3K27ac (Active Motif, #39133) and anti-IgG (Diagenode, C15410206). The eluted DNA fragments were analysed by qPCR as described above. H3K27ac ChIP was performed as described above but cells were fixed for 5 min in 1 % formaldehyde. The primers for the target regions are listed in the following table:

Target	Forward primer	Reverse primer
Gria1	GGTTAGGGTGTGACAAATGGG	TCCTGTTCTGTGGGCTCA TTC
Gria2	GGCACAATTCACACAGGGAC	AAGGGTTAACAGGAGAC GAGG
Bdnf	TCTGATGCAATTCCAAGCGTG	AATCTCCCAGTTCTGCGT TCA
Syt1	CACTTCCTGGACAGCTTAGCA	TTTGAGCCCTGTGACTGT CAG
cluster1_prkarb_intron	CTTGGGAAGAGTGTGCTT GTG	TAAACAGACCCAGCCAG CAC
cluster2_intergenic	TCCATTTCCTTCTCACTGGGC	ATCACCGCGCAATTGTTA ACC
cluster2_Rbms3_intron	GGCAGGCAGAATTTCAGACAC	AGCCATGCCCAAAAGTGA GAT
cluster 3_intergenic	AGAAACCGCTTGCAGTACTGA	CACAACGGATGCTAAGTA GGC
K27ac_up_prox1_upstream	CACAACTCGTTCTGCTTTGGA	AATATCAGACTTAGCGGC CCC
H3K27ac_down_intergenic	TGGAATCAAACCTCTCTCCAGC	TGGCCCTTGTTCCAATAT CCA

Co-Immunoprecipitation (Co-IP)

Tissue was lysed in lysis buffer (300 mM NaCl, 20 mM Tris, 1 mM EDTA, 0.5% Nonidet-40 (NP-40), pH 7.4) supplemented with protease inhibitor (Sigma-Aldrich) and incubated for 30 min on ice, triturating every 10 min 20 times. After a 10 min centrifugation at 13000 rpm, the supernatant was collected. The salt and NP-40 concentrations were brought down to 100 mM and 0.15%, respectively, using equilibration buffer (20 mM Tris, 1mM EDTA, pH 7.4). Protein concentrations were determined with Bradford reagent (Bio-Rad). 10% input was saved and equal amounts of protein were used for all Co-IPs. Protein G Dynabeads (10004D, Thermo Scientific) were washed once with Co-IP buffer (100 mM NaCl, 20 mM Tris, 1 mM EDTA, 0.15% NP-40 pH 7.4), and incubated with Co-IP antibodies at 4°C overnight. The following antibodies were used: anti-HDAC2 (#57156, Cell Signaling), anti-NEUROD1 (#Ab109224,

Abcam), anti-SIRT1 (#8469, Cell Signalling), anti-HDAC1 (#sc-81598, Santa Cruz), IgG (#C15410206 or #C15400001, Diagenode). Antibody-coupled beads were washed once with co-IP buffer before co-IP. Cell lysates were blocked with Protein G Dynabeads for 1 h at 4°C for preclearing, subsequently transferred to antibody-coupled beads, and incubated overnight at 4°C in rotation. Antibody-coupled beads were washed 3 times with Co-IP buffer before they were resuspended in 1X Laemmli sample buffer. Protein-antibody complexes were eluted by incubating the beads at 70ºC for 10 min at 550 rpm. 10% input and the complete Co-IP sample were used for immunoblotting.

Immunoblotting

Co-IP or protein samples were loaded on 8% or 10% SDS-polyacrylamide gels and run at 120 V for 1.5 h in Tris-Glycine running buffer. Proteins were transferred to PVDF membranes (Trans-blot Turbo Transfer Pack, Bio-Rad) using the Trans-blot Turbo Transfer System (Bio-Rad) following the manufacturer's instructions. Membranes were blocked with 5% BSA in TBS (Tris buffered saline) for 1 h. The following antibodies were used immunoblots: anti-FOXG1 (detecting monomers and dimers; anti-FOXG1, #61211, Active Motif), anti-HA-tag (#3724, Cell Signaling), anti-GAPDH (#ab8245, Abcam) or anti-H3 (#Ab1791-100, Abcam) diluted in 5% BSA in TBST (Tris buffered saline with 0.1% Tween-20). Membranes were washed 3 times with TBST and incubated with secondary antibody anti-rabbit HRP (1:10000 dilution in 5% BSA in TBST) or Tidyblot (Biorad, 1:5000 dilution) for 1 h. Membranes were washed twice with TBST and once with TBS before being developed using SuperSignal™ West Femto Maximum Sensitivity Substrate (Thermo Scientific). Membranes were imaged using LAS ImageQuant System (GE Healthcare). Protein levels were normalized and quantified using ImageStudio Lite (v5.2) software.

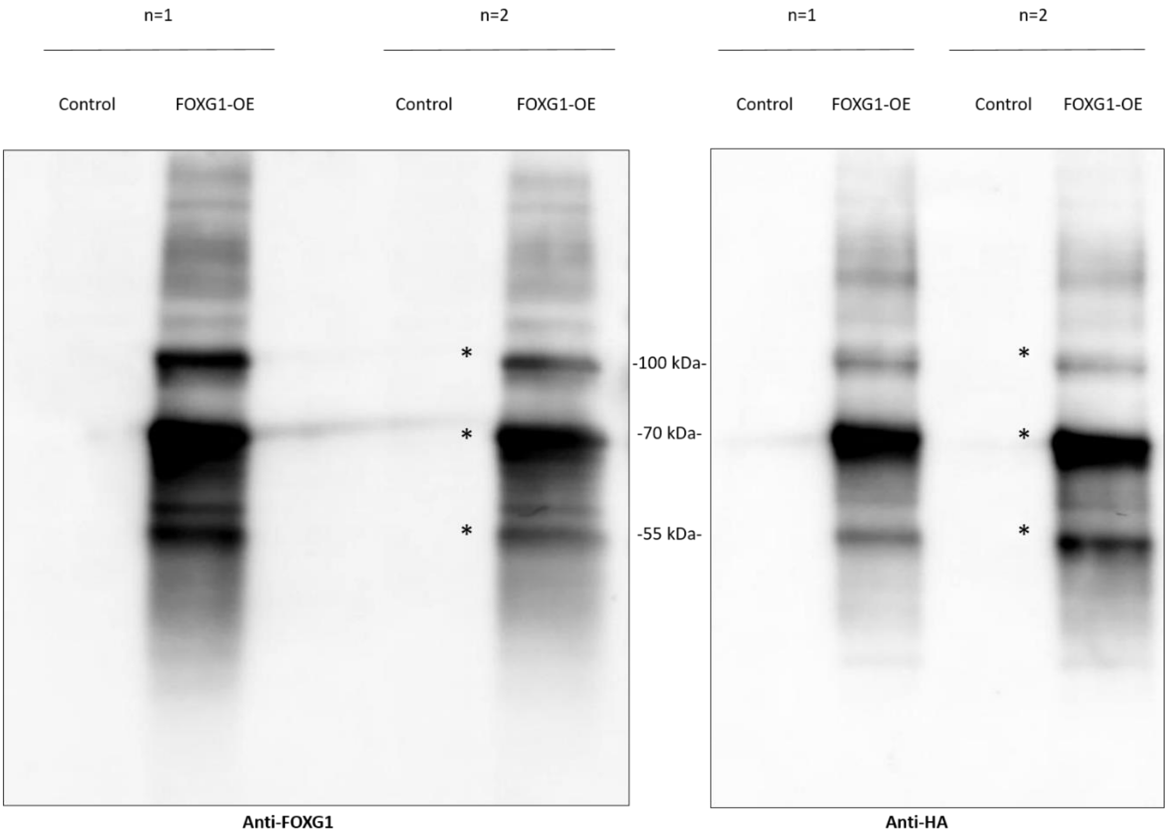

Figure i: Immunoblots confirming FOXG1 antibody specificity in samples from FOXG1 overexpression in N2A cells (n=2). Left: anti-FOXG1 (#61211, Active Motif); Right: anti-HA (#3724, Cell Signaling). FOXG1 bands are at 110, 70 and 55 kDa.

Tissue sections, in situ hybridization and immunostaining

PFA fixed brains were embedded in TissueTec (SAKURA) cut in 14 µm sections and mounted on SuperFrost Plus Microscope slides (Thermo Scientific). Probes for *in situ* hybridization were made by cloning PCR products into pGemTeasy (Promega). 1 µg of the linearized plasmid was transcribed *in vitro* using NTP labelling mix and T7 or SP6 RNA Polymerase, followed by purification with mini Quick spin RNA columns (Roche). Probes were diluted in hybridization buffer in 1:500 or 1:1000 ratio and incubated on the sections overnight at 68°C. After washing and blocking in lamb serum in MABT buffer (5X MAB, 0.1% Tween 20), sections were incubated with Anti-DIG-AP (Roche) at 4°C overnight. After washing, sections were developed with NBT/BCIP (Roche) overnight and mounted. Bright field images were obtained using an Axioplan M2 microscope (Zeiss). Immunofluorescence was performed as described previously (3, 4) using antibodies against SATB2 (Abcam, ab92446), CTIP2 (Abcam, ab18465) and ZBTB20 (Sigma, HPA016815). Images were obtained using a primary magnification of 20x, and stitched together to ensemble the complete hippocampus using an Axioplan M2 fluorescent microscope (Zeiss) equipped with an Apotome.2 module.

DiI tracing

Brains from 7 week old mice were dissected and immersion-fixed in 4% paraformaldehyde in 0.1 M phosphate buffer for 2-3 days and horizontal tissue sections of 2-3 mm thickness were cut by a razor blade. Small crystals of DiI (1,1'-dioctadecyl-3,3,3',3'-tetra-methylindocarbocyanine perchlorate) mounted on the tip of a glass micropipette were placed into different regions of the hippocampal formation under visual control. The brain sections

were then stored for 5-6 weeks in the fixative solution at RT and in the dark to allow the labelling of fiber pathways. Next, sections were washed in PB and sliced into 100 µm thick vibratome sections and mounted on glass slides for fluorescence microscopy. Digital images were made using either Axioplan M2 (Zeiss) or ApoTome 1 (Zeiss).

N2A cell culture

Mouse neuroblastoma cell line, Neuro-2a (N2A) was cultured in Dulbecco's modified Eagle's medium (DMEM, Thermo Scientific) supplemented with 10% fetal bovine serum (FBS, Thermo Scientific), 1% non-essential amino acids (NEAA, Thermo Scientific), 1% L-glutamine, and 1% penicillin, streptomycin, and neomycin (PSN, Thermo Scientific). Cells were maintained at 37°C, 95% relative humidity and 5% CO_2.

FOXG1 overexpression in N2A cells

N2A cells were maintained and transfected as described above. 25.000 cells/well were seeded in 6 well plates. 24 hours after seeding, cells were transfected with a total amount of 2.5 µg plentiIII-FoxG1-HA-2A-GFP (accession for the Foxg1 insert sequence: NM_001160112) or the empty vector control plentiIII-HA-2A-GFP plasmids using Lipofectamine LTX (Thermo Scientific) according to the instructions of the manufacturer. 48 hours post-transfection, cells were washed twice with cold PBS, collected, snap frozen, and stored at -80 ˚C.

Dual Luciferase Reporter Assay

50.000 N2A cells/well were seeded to 24-well plates overnight and double-transfected the following morning with 2.5 µg pMirGlo luciferase reporter constructs and pLenti-III overexpression plasmid DNA using Lipofectamine LTX (Thermo Scientific) reagent. pMirGlo reporters contained inserts with *Ncald* or *Ldb2* regulatory regions with or without targeted ablation of Fkh or bHLH/E-Box motifs. pLenti-III overexpression constructs contained inserts with a sequence coding for the intact FOXG1 protein (FOXG1 OE) or a protein bereft of the Fkh-binding domain (Foxg1 ΔFKH) (30). Medium was changed after 24 h. 48 h after transfection, cells were washed once with 1X PBS and incubated with 1X passive lysis buffer (Promega) at RT for 20 min, gently agitating. Lysates were loaded in triplicate to 96-well microtiter plates and luminescence was measured in a GloMax 96 microplate luminometer (Promega). Gene expression was measured by firefly luciferase activity (LAR II; Promega) normalized against the renilla luciferase signal (Stop & Glo; Promega), which served as a control. Background noise was filtered out of both signals prior to calculation by subtracting luminosity measured before enzyme injection. All promoter constructs were tested with at least three biological replicates.

Luciferase reporter sequences:

Ldb2

TTAACATGATGTTAATTATTTGTAAATTGTATGTTTGGATACACATTACTTGAGAGGCATATGTTATATTCAA

Ldb2, Fkh deleted

TTAACATGATGTTAATTAGTAAATTGTATGGGATACACATTACTTGAGAGGCATATGTTATATTCAA

Ldb2, E-box deleted

TTAACATGATGTTAATTATTTGTAAATTGTATGTTTGGATACACATTACTTGAGAGGCAGT TATATTCAA

Ncald

GGAACAATGCAACCAAATATGGACATTCTATTTAGAAACAGTAATGTTAGCATAACAAGG GAGCTTAAAAAAAGAAAAAGCTAAATAAATATTAAACAGATGGGTGGTCAAATGTTGTTT TTCTTTTTAAGCACAAAGCCTTAATCTAAAGCCAAGCAG

Ncald, Fkh deleted

GGAACAATGCAACCAAATATGGACATTCTAAGCAGTAATGTTAGCATAACAAGGGAGCT TAAAAAAAGAAAAAGCTTATTCAGATGGGTGGTCAAATGTTGTTTTTCTTTTTAAGCACA AAGCCTTAATCTAAAGCCAAGCAG

Ncald, E-box deleted

GGAACAATGCAACCAAATATGGACATTCTATTTAGAAACAGTAATGTTAGCATAACAAGG GAGCTTAAAAAAAGAAAAAGCTAAATAAATATTAAACAGGGTGGTCAAATGTTGTTTTTC TTTTTAAGCACAAAGCCTTAATCTAAAGCCAAGCAG

Ncald, Fkh & E-box deleted

GGAACAATGCAACCAAATATGGACATTCTAAGCAGTAATGTTAGCATAACAAGG

GAGCTTAAAAAAAGAAAAAGCTTATTCAGGGTGGTCAAATGTTGTTTTTCTTTTT

AAGCACAAAGCCTTAATCTAAAGCCAAGCAG

RNA-seq

Total RNA extracted from mouse hippocampi or DIV11 primary hippocampal neurons were used for RNA-seq. Libraries were prepared using the TruSeq total RNA sample preparation kit from Illumina, following the manufacturer’s instructions. The procedure included depletion of rRNA prior double-stranded cDNA synthesis and library preparation. Samples were sequenced on Illumina HiSeq3000 as paired-end 101 bp reads.

**Datasets**	**N**
E18.5: Foxg1$^{cre/+}$/Foxg1$^{+/+}$	3
6 week old: Foxg1$^{cre/+}$/Foxg1$^{+/+}$	2
DIV11 neurons: Foxg1$^{cre/+}$/Foxg1$^{+/+}$	3
DIV11 neurons: shFoxg1/ shControl	4
DIV11 neurons: shFoxg1+TSA/ shControl+TSA	4

For cultured neurons, each replicate (n) came from independent cultures of different litters. For *in vivo* samples, each replicate was from independent mice.

ChIP-seq

In vivo hippocampal tissue samples
CA and DG regions of the hippocampus were dissected separately and snap frozen in liquid nitrogen. FOXG1 ChIP-Seq from frozen tissue samples (n=1-2) was performed by Active Motif Services (Carlsbad, CA). In brief, tissue was submerged in PBS + 1% formaldehyde, cut into small pieces and incubated at room temperature (RT) for 15 min. Fixation was stopped by the addition of 125 mM glycine (final concentration). The tissue pieces were subsequently treated with a TissueTearer and finally spun down and washed 2 times in PBS. Chromatin was

isolated by the addition of lysis buffer, followed by disruption with a Dounce homogenizer. Lysates were sonicated and the DNA sheared to an average length of 300-500 bp. Genomic DNA (input) was prepared by treating aliquots of chromatin with RNase, proteinase K and heat for de-crosslinking, followed by ethanol precipitation. Pellets were resuspended and the resulting DNA was quantified on a NanoDrop spectrophotometer. Extrapolation to the original chromatin volume allowed quantitation of the total chromatin yield. An aliquot of chromatin (12 µg) was precleared with protein A agarose beads (Invitrogen). Genomic DNA regions of interest were isolated using 4 µg of antibody against FOXG1 (Active Motif, cat# 61211, lot# 34711001). Complexes were washed, eluted from the beads with SDS buffer, and subjected to RNase and proteinase K treatment. Crosslinks were reversed by incubation overnight at 65ºC, and ChIP DNA was purified by phenol-chloroform extraction and ethanol precipitation.

Quantitative PCR (qPCR) reactions were carried out in triplicate on specific genomic regions using SYBR Green Supermix (Bio-Rad). The resulting signals were normalized for primer efficiency by carrying out qPCR for each primer pair using input DNA.

Illumina sequencing libraries were prepared from the ChIP and input DNAs by the standard consecutive enzymatic steps of end-polishing, dA-addition, and adaptor ligation. Steps were performed on an automated system (Apollo 342, Wafergen Biosystems/Takara). After a final PCR amplification step, the resulting DNA libraries were quantified and sequenced on Illumina's NextSeq 500 (75 nt reads, single end).

In vitro primary hippocampal neuron samples

Wild-type or virally transduced primary hippocampal neurons were fixed and collected for subsequent FOXG1 or NEUROD1 (n=1-2) ChIP-seq experiments according to the instructions of the servicing company (Active Motif, Carlsbad, CA). Briefly, cells were fixed by adding 1/10 volume formaldehyde solution (11% formaldehyde, 0.1 M NaCl, 1 mM EDTA pH=8.0, 50 mM HEPES pH=7.9) directly to the medium and incubated at RT with agitation. Fixation was stopped by adding 1/20 volume 2.5 M Glycine solution (Roth, Germany) and incubating for 5 min at RT. Cells were scraped from the plates and transferred into conical tubes, and centrifuged at 800 rpm at 4°C for 10 min. Cells were resuspended and washed twice in chilled PBS-Igepal solution (0.5% Igepal CA-360 (Sigma) in 1X PBS pH 7.4 (Gibco), and a third time in PBS-Igepal-PMSF (phenylmethylsulfonyl fluoride) solution (0.5% Igepal, 1 mM PMSF (Sigma) in 1X PBS pH 7.4). The supernatant was completely removed and cell pellets were snap frozen in liquid nitrogen and stored at -80°C until following ChIP-seq experiments by Active Motif, as described above.

ChIP-seq libraries were generated from ChIP-DNA using a custom Illumina library type on an automated system (Apollo 342, Wafergen Biosystems/Takara). ChIP-seq libraries were sequenced on Illumina NextSeq 500 as single-end 75bp reads (Active Motif, Carlsbad, CA).

RELACS ChIP-seq

Restriction enzyme-based labeling of chromatin *in situ* (RELACS) was used for H3K27ac (n=2) and H3K4me3 (n=2) ChIP-sequencing (5). DIV11 primary hippocampal neurons with FOXG1 or luciferase KD were fixed in 1% formaldehyde for 15 min. The reaction was stopped with 125 mM glycine for 5 min, followed by two-time DPBS +PIC washes. Cell nuclei were isolated following the NEXSON protocol (treatment time 20 s) and permeabilized with 0.5% SDS (2). Chromatin was digested *in situ* using five units of restriction enzyme CviKI-1 (NEB, R0710S) every 100.000 nuclei and RELACS custom barcodes (4 bp UMI + 8 bp RELACS barcode) were ligated to end-repaired and A-tailed chromatin using components from NEBNext Ultra II DNA Library Prep Kit for Illumina (NEB) (2).

The barcoded chromatin fragments were extracted by sonication for 5 min using these parameters: peak power 105 W, 2% duty factor, 200 cycles/burst (Covaris microtubes (520185), Covaris E220 Focused-ultrasonicator). A single immunoprecipitation (IP) reaction for all pooled samples was carried out on SX-8G Compact IP-Star platform (Diagenode) following Arrigoni et al. (5). Immunoprecipitated chromatin was used for NGS library preparation (NEBNext Ultra II DNA Library Prep Kit for Illumina, NEB). Libraries were sequenced using HiSeq 3000 (Illumina) as 75 bp reads.

ATAC-seq

ATAC-seq was performed on DIV11 primary hippocampal neurons after FOXG1 or luciferase KD (n=2) following the protocol of Buenrostro et al., 2015 (5). Briefly, cells were washed with PBS and detached by 5 min 0.05% trypsin incubation. Dissociation was stopped with 10% FBS and cells were collected and centrifuged for 5 min at 500 rpm at 4°C. After one time wash with ice-cold PBS, cells were counted and separated into $5x10^5$cells/replicate/condition. Cells were resuspended in lysis buffer (10 mM Tris-HCl pH 7.4, 10 mM NaCl, 3 mM MgCl2, 0.1% Igepal Ca-630) and immediately centrifuged for 10 min at 500 rpm at 4°C. Transposition, PCR amplification and DNA library preparation were done using Nextera DNA library prep kit according to the manufacturer's instructions (Illumina). DNA was purified and eluted using MinElute PCR purification kit (Qiagen). Libraries were sequenced using HiSeq 3000 (Illumina) (paired-end 100bp reads).

Statistics

GraphPad Prism software (version 9.1.1) was used for statistical analyses. Two-way ANOVA followed by Tukey multiple comparisons was used for the analyses of dual luciferase assay and *in vivo* TSA qRTPCR experiment. One-way ANOVA or unpaired t-test was used for immunoblots and qRTPCR anlaysis. Fisher's exact test was used to test the enrichment of DEGs in clusters and datasets using the *GeneOverlap* (6) package in R Bioconductor (v. 3.8). *bedtools FisherBed* (7) was used to test the overlap between *in vivo* and *in vitro* FOXG1 peak datasets.

Artwork

The illustrations were created with Biorender.com.

Bioinformatics, data repository and analyses of public databases

The "Differential Search" tool of the Allen Brain Atlas (8) was used to define field-specific gene expression. The respective subfield was set as target structure and the other fields as contrast structure. The emerging list was visually inspected with ISH datasets to confirm selective expression in the field as well as overall expression. Assignment to a field was performed according to a clearly visible ISH signal. From this list, we compiled the subset of genes tested in qRTPCR analysis.

The sequencing data from RNA-, ATAC-, RELACS-, and wildtype ChIP-seq were processed with *snakePipes* (v. 1.1.1) (9). Summary of quality control is available at https://github.com/Vogel-lab/Integrative-multi-omics-analyses-of-FOXG1-functions. Mapping was performed on mouse genome build mm10 (GRCm38). For ChIP-Seq and ATAC-seq, high quality and uniquely mapping reads were retained (mapq > 5). RELACS custom barcodes were designed with integrated UMI, so duplicate removal was performed using *UMITools* (10), while standard deduplication was applied for ATAC-seq reads. For ChIP-seq and ATAC-seq

data, *snakePipes* also provided candidate peak regions using *MACS2* (v. 2.2.6) using default parameters.

Differential expression analysis for RNA-seq was done using *DESeq2* (v. 1.22.1) (11) on count matrices output from *snakePipes* (*featureCounts*, v. 1.6.4) (12). A linear model controlling for batch effects (e.g., ~batch + treatment or ~ batch + condition) was used and apeglm $\log_2$(Fold Change) shrinkage was applied.

Differential ChIP-seq and ATAC-seq analyses were performed on consensus peak sets, coverage was computed using *multiBamSummary (deeptools)* (13) and differential regions were identified via *csaw* (v. 3.13) (14).

The sequencing data from FOXG1 and NEUROD1 ChIP-seq after NEUROD1 or FOXG1 KD were uploaded to the Galaxy web platform, and the public server at usegalaxy.eu was used to analyze the data (15). Same parameters were applied for quality control and mapping as the SnakePipes analyses. Peaks were called using *MACS2 callpeak* (Galaxy Version 2.1.1.20160309.6). Coverage was computed using *multiBamSummary*, and bam files were normalized by *bamcompare* and *bigwigcompare* (*deeptools*, Galaxy Version 3.3.2.0.0) (13). Differential binding regions of NEUROD1 were computed using *DiffBind* (Galaxy Version 2.10.0) (16).
All metaprofiles and heatmaps of ChIP- and ATAC-seq signals were generated with *deeptools* (Galaxy Version 3.3.2.0.0) (13). All ChIP- and ATAC-seq peaks were annotated and visualized using *ChIPSeeker* (Galaxy Version 1.18.0) (17).

Upset plots of peak interval overlaps was done using *Intervene* (Galaxy Version 0.6.5) (18), and the statistics analysis of the overlaps was done using *bedtools FisherBed* (Galaxy Version 2.30.0) (7).

GimmeMotifs (v.0.13.1 and v.0.16.0) was used for motif enrichment and differential motif analysis (19).

GO enrichment and differential GO-term analyses were performed using *clusterProfiler* (v. 4.2.2) (20).

Visualizations were done in Galaxy, R (v. 4.1) and Python (v. 3.6). Heatmaps were plotted using *heatmap2* (Galaxy Version 3.0.1) or pheatmap (21), volcano plots using *EnhancedVolcano* (BioConductor, v. 3.13) (22), violin plots using *ggplot2* (v. 3.3.5) (23), and Venn diagrams using *ggVenn* (24) and *VennDiagram* (25) packages. Fisher's exact test was applied to DEGs and clustered peak overlaps using the *GeneOverlap* (v. 3.8) (6).

Hipposeq

Hipposeq (26) was accessed at http://hipposeq.janelia.org/ choosing the dorsal-ventral survey of hippocampal principal cells. Dorsal and ventral populations of CA1, CA2 and CA3 were chosen and the gene names were entered as a list using the standard settings of the database, and the count matrix was normalized as transcripts per million (TPM). The set of genes with significant increase or decrease in expression from our RNA-seq were intersected with the TPM normalized count matrix from hipposeq. The genes found in the intersection were clustered using hierarchical clustering on the row-wise Z score.

STRING database

STRING database (27) was used to explore known and predicted protein-protein interactions of FOXG1 in *Mus musculus*. Data from experiments and databases were used with medium confidence (0.4) for the interaction scores. Nodes annotated interactor proteins, while edges

connected interacting proteins. Line thickness indicated the confidence of interaction. The interactome layout was reorganized in Cytoscape (v 3.8.2) to depict direct interactions of FOXG1 with HDACs ad HATs.

Supplementary Figures:

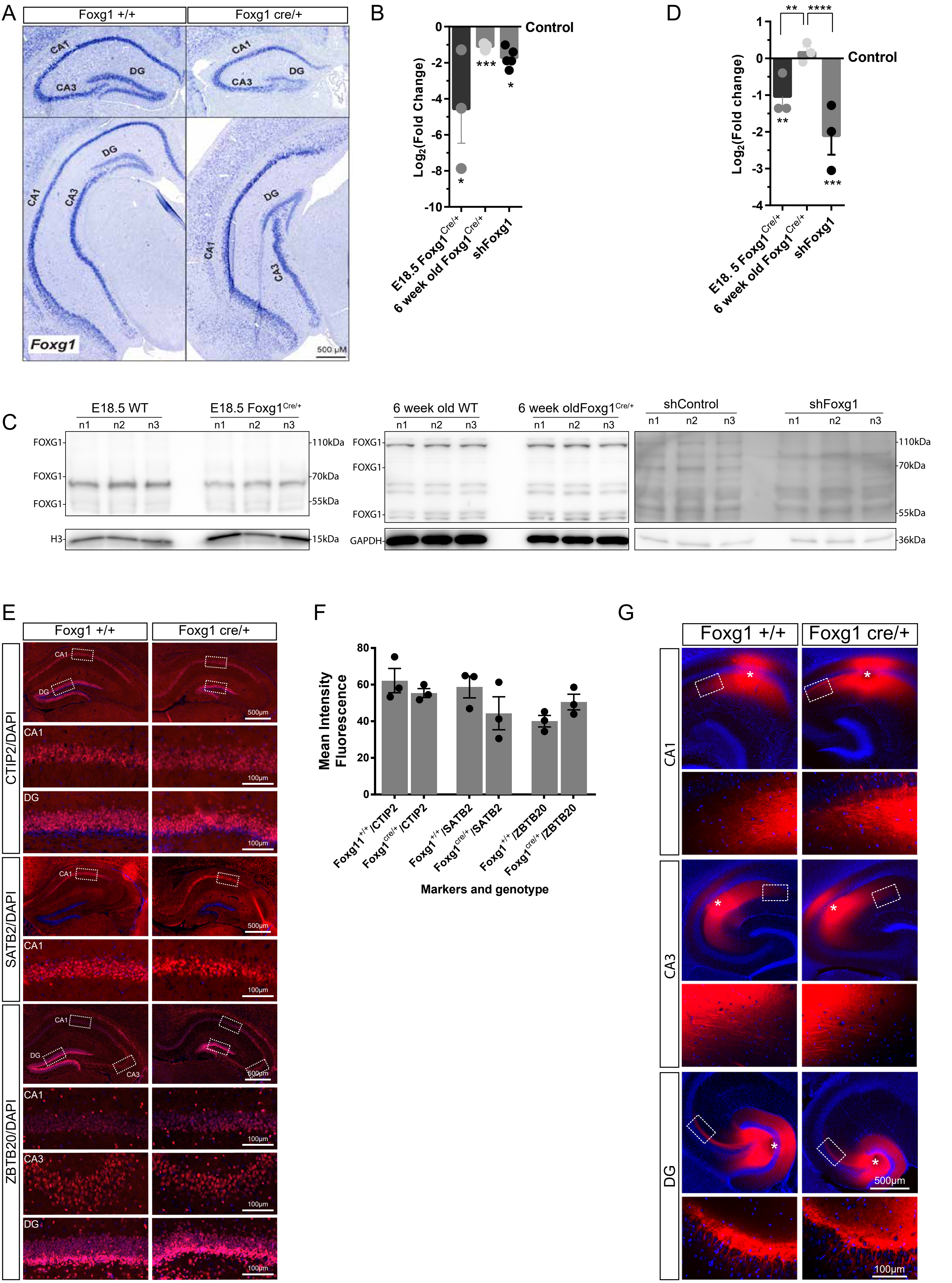

A
Foxg1 +/+
Foxg1 cre/+
CA1
CA3
DG
Foxg1
500 µM
B
Control
Log2(Fold Change)
E18.5 Foxg1Cre/+
6 week old Foxg1Cre/+
shFoxg1
D
Control
Log2(Fold change)
E18. 5 Foxg1Cre/+
6 week old Foxg1Cre/+
shFoxg1
C
E18.5 WT
E18.5 Foxg1Cre/+
6 week old WT
6 week oldFoxg1Cre/+
shControl
shFoxg1
n1 n2 n3
FOXG1
H3
GAPDH
110kDa
70kDa
55kDa
15kDa
36kDa
E
Foxg1 +/+
Foxg1 cre/+
CTIP2/DAPI
SATB2/DAPI
ZBTB20/DAPI
CA1
CA3
DG
500µm
100µm
F
Mean Intensity Fluorescence
Foxg11+/+/CTIP2
Foxg1cre/+/CTIP2
Foxg1+/+/SATB2
Foxg1cre/+/SATB2
Foxg1+/+/ZBTB20
Foxg1cre/+/ZBTB20
Markers and genotype
G
Foxg1 +/+
Foxg1 cre/+
CA1
CA3
DG
500µm
100µm

Supplementary Figure Legends:

Figure S1: Characterization of gene expression levels of FOXG1$^{cre/+}$ adult mouse hippocampus and upon FOXG1 KD in primary hippocampal neurons.

(A) Expression pattern of *Foxg1* in the 6 week old Foxg1$^{+/+}$ (left, control) and Foxg1$^{cre/+}$ (right) mouse hippocampus was detected by in situ hybridization. *Foxg1* was expressed in both the dentate gyrus (DG), and in the granule cells of the CA fields. Foxg1$^{cre/+}$ mice had smaller hippocampus, although the CA and DG fields were preserved (n=3). **(B)** qRTPCR quantification of Foxg1 mRNA levels in E18.5 and 6 week old Foxg1$^{cre/+}$ hippocampus compared to shFoxg1 knockdown of FOXG1 in DIV11 hippocampal neurons compared to control. Y-axis: LFC. **(C)** Immunoblots of FOXG1 of *in vivo* E18.5 and 6 weeks old (Foxg1$^{+/+}$, Foxg1$^{cre/+}$), and *in vitro* (shFoxg1 and shLuciferase) samples (n=3). FOXG1 bands are at 110, 70 and 55 kDa. **(D)** Quantification of the immunoblot in (B). Y-axis: LFC. One-way ANOVA with Tukey's multiple comparisons *: $p<0.05$, **: $p<0.01$, ***: $p<0.001$. **(E)** Immunostainings of 3 week old brains of *Foxg1*$^{cre/+}$ and *Foxg1*$^{+/+}$ for SATB2, CTIP2 and ZBTB20. The first row of images in each panel shows an overview of the hippocampus. The lower rows represent a magnification of the indicated regions in the overviews (n= 3). **(F)** Quantification of SATB2, CTIP2 and ZBTB20 stainings in (B) showed no significant difference between Foxg1$^{cre/+}$ and Foxg1$^{+/+}$. Y-axis shows the mean intensity of fluorescence and x-axis shows the markers and the genotypes that are quantified (dark gray: Foxg1$^{+/+}$, light gray: Foxg1 $^{cre/+}$). Unpaired t-test. **(G)** DiI tracing in CA1, CA3, and DG in 7 week old mice shows that haploinsufficiency of *Foxg1* did not affect intra-hippocampal connectivity (Foxg1$^{+/+}$ n=3, Foxg1$^{cre/+}$ n=5). CA: cornu ammonis, DG: dentate gyrus. Scale bars as indicated within the figures.

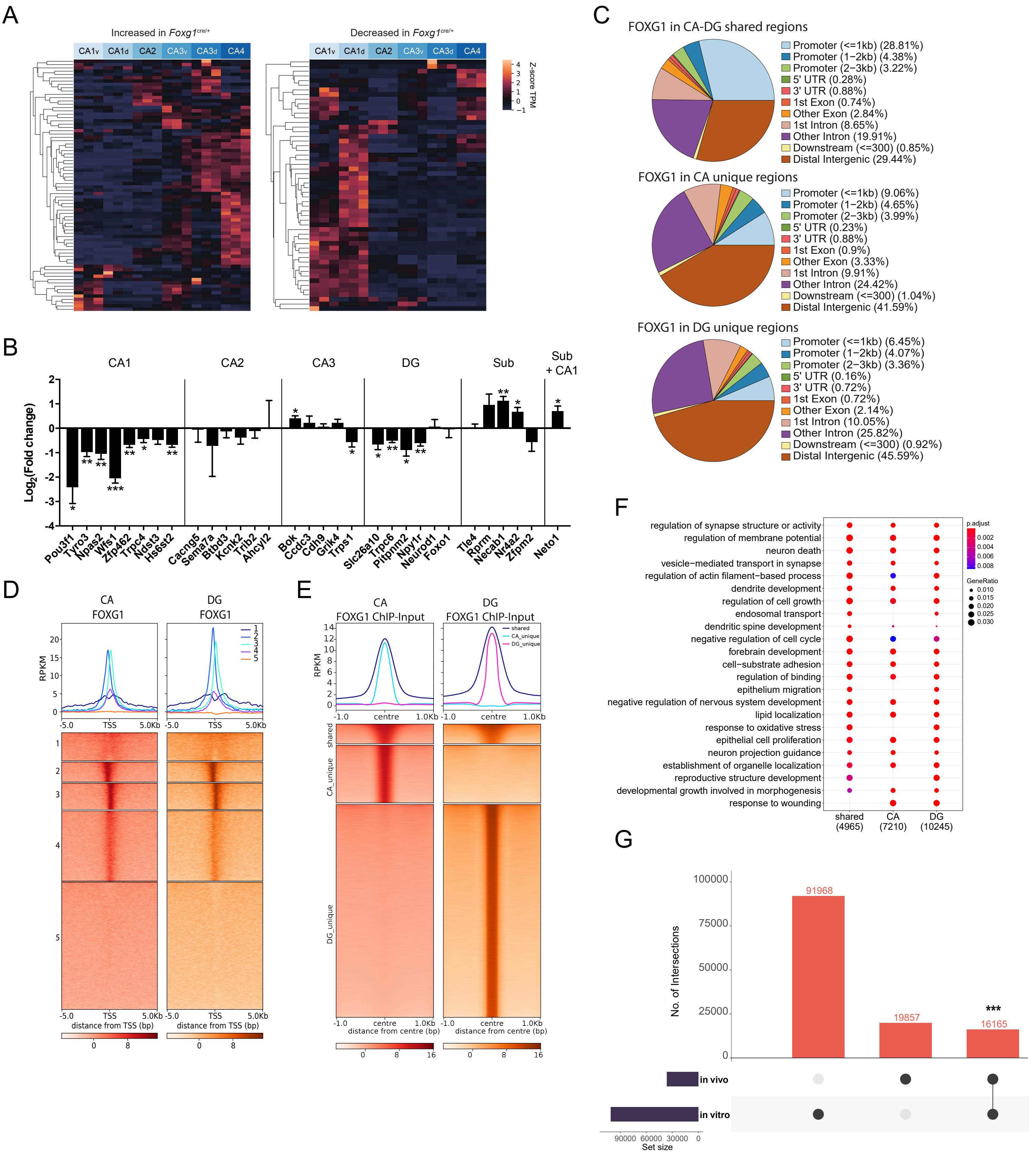
A
Increased in Foxg1cre/+
Decreased in Foxg1cre/+
CA1v CA1d CA2 CA3v CA3d CA4
Z-score TPM
C
FOXG1 in CA-DG shared regions
Promoter (<=1kb) (28.81%)
Promoter (1−2kb) (4.38%)
Promoter (2−3kb) (3.22%)
5' UTR (0.28%)
3' UTR (0.88%)
1st Exon (0.74%)
Other Exon (2.84%)
1st Intron (8.65%)
Other Intron (19.91%)
Downstream (<=300) (0.85%)
Distal Intergenic (29.44%)
FOXG1 in CA unique regions
Promoter (<=1kb) (9.06%)
Promoter (1−2kb) (4.65%)
Promoter (2−3kb) (3.99%)
5' UTR (0.23%)
3' UTR (0.88%)
1st Exon (0.9%)
Other Exon (3.33%)
1st Intron (9.91%)
Other Intron (24.42%)
Downstream (<=300) (1.04%)
Distal Intergenic (41.59%)
FOXG1 in DG unique regions
Promoter (<=1kb) (6.45%)
Promoter (1−2kb) (4.07%)
Promoter (2−3kb) (3.36%)
5' UTR (0.16%)
3' UTR (0.72%)
1st Exon (0.72%)
Other Exon (2.14%)
1st Intron (10.05%)
Other Intron (25.82%)
Downstream (<=300) (0.92%)
Distal Intergenic (45.59%)
B
CA1 CA2 CA3 DG Sub Sub + CA1
Log2(Fold change)
Pou3f1 Tyro3 Npas2 Wfs1 Zfp462 Trpc4 Ndst3 Hs6st2 Cacng5 Sema7a Btbd3 Kcnk2 Trib2 Ahcyl2 Bok Ccdc3 Cdh9 Grik4 Trps1 Slc26a10 Trpc6 Pitpnm2 Npy1r Neurod1 Foxo1 Tle4 Rprm Necab1 Nr4a2 Zfpm2 Neto1
D
CA FOXG1
DG FOXG1
RPKM
-5.0 TSS 5.0Kb
distance from TSS (bp)
E
CA FOXG1 ChIP-Input
DG FOXG1 ChIP-Input
shared
CA_unique
DG_unique
-1.0 centre 1.0Kb
distance from centre (bp)
F
regulation of synapse structure or activity
regulation of membrane potential
neuron death
vesicle−mediated transport in synapse
regulation of actin filament−based process
dendrite development
regulation of cell growth
endosomal transport
dendritic spine development
negative regulation of cell cycle
forebrain development
cell−substrate adhesion
regulation of binding
epithelium migration
negative regulation of nervous system development
lipid localization
response to oxidative stress
epithelial cell proliferation
neuron projection guidance
establishment of organelle localization
reproductive structure development
developmental growth involved in morphogenesis
response to wounding
shared (4965) CA (7210) DG (10245)
p.adjust
GeneRatio
G
No. of Intersections
91968
19857
16165
***
in vivo
in vitro
Set size

Figure S2: *Foxg1* haploinsufficiency affects gene expression in the CA fields and DG *in vivo*, but its cistrome is comparable *in vivo* and *in vitro*.

(A) Genes that showed increased expression (left panel) in hippocampus of 6 week old Foxg1$^{cre/+}$ mice show relatively high expression in CA3/4. Genes that showed decreased expression (right panel) in hippocampus of 6 week old Foxg1$^{cre/+}$ mice show relatively high expression in CA1, as assessed with *Hipposeq*. **(B)** Expression analysis of marker genes for hippocampal subfields shows that CA1 and DG are most prominently affected after KD of FOXG1. qRTPCR analysis of selected marker genes for hippocampal subfields in hippocampus of 6 week old Foxg1$^{cre/+}$ mice compared to their respective wild type littermates shows that genes with high expression in CA1 or DG mostly decreased after KD of FOXG1. CA: cornu ammonis, DG: dentate gyrus, sub: subiculum. All qRTPCR data are represented as mean± SEM, n=3-5, *: p<0.05, **: p<0.01, ***p<0.001, unpaired Student's t-test. **(C)** Genomic distribution of FOXG1 peaks in CA-DG shared, CA-unique, and DG-unique binding regions represented as pie charts. **(D)** K-means clustering (k=5) of FOXG1 enrichment in CA (red) and DG (orange) regions found 5 Kb up/downstream of TSS of protein coding genes. Data is normalized by sequencing depth and input control. The metaprofiles (top) show the average reads (RPKM) of each cluster. **(E)** Heatmap showing FOXG1 enrichment at binding sites clustered into shared, CA-unique, and DG-unique regions. Data normalization and metaprofiles (top) as in D. **(F)** GO-terms were compared among clusters derived from E. Scales of gene ratio and adjusted p-value are reported at the top right. Total number of genes per cluster are on the x-axis. Threshold for enrichment: p < 0.01 **(G)** Upset plot showing the significant overlap between *in vivo* (CA_DG combined) and *in vitro* (N1) FOXG1 peaks. ***p<0.001, *bedtools FisherBed.* (CA: n=2, DG: n=1, in vitro: n=1).

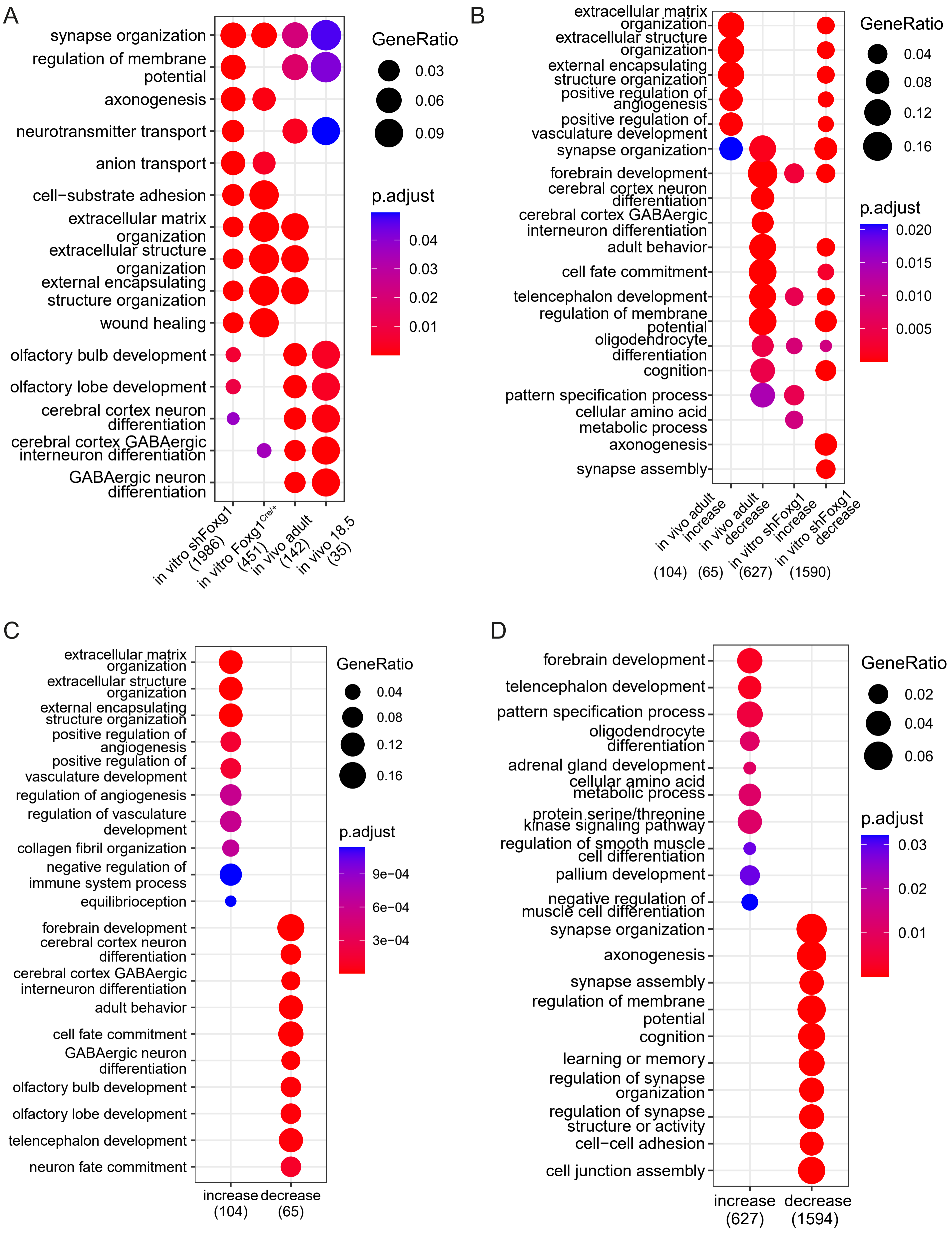
A
synapse organization
regulation of membrane potential
axonogenesis
neurotransmitter transport
anion transport
cell−substrate adhesion
extracellular matrix organization
extracellular structure organization
external encapsulating structure organization
wound healing
olfactory bulb development
olfactory lobe development
cerebral cortex neuron differentiation
cerebral cortex GABAergic interneuron differentiation
GABAergic neuron differentiation
in vitro shFoxg1 (1986)
in vitro Foxg1Cre/+ (451)
in vivo adult (142)
in vivo 18.5 (35)
GeneRatio
0.03
0.06
0.09
p.adjust
0.04
0.03
0.02
0.01
B
extracellular matrix organization
extracellular structure organization
external encapsulating structure organization
positive regulation of angiogenesis
positive regulation of vasculature development
synapse organization
forebrain development
cerebral cortex neuron differentiation
cerebral cortex GABAergic interneuron differentiation
adult behavior
cell fate commitment
telencephalon development
regulation of membrane potential
oligodendrocyte differentiation
cognition
pattern specification process
cellular amino acid metabolic process
axonogenesis
synapse assembly
in vivo adult increase (104)
in vivo adult decrease (65)
in vitro shFoxg1 increase (627)
in vitro shFoxg1 decrease (1590)
GeneRatio
0.04
0.08
0.12
0.16
p.adjust
0.020
0.015
0.010
0.005
C
extracellular matrix organization
extracellular structure organization
external encapsulating structure organization
positive regulation of angiogenesis
positive regulation of vasculature development
regulation of angiogenesis
regulation of vasculature development
collagen fibril organization
negative regulation of immune system process
equilibrioception
forebrain development
cerebral cortex neuron differentiation
cerebral cortex GABAergic interneuron differentiation
adult behavior
cell fate commitment
GABAergic neuron differentiation
olfactory bulb development
olfactory lobe development
telencephalon development
neuron fate commitment
increase (104)
decrease (65)
GeneRatio
0.04
0.08
0.12
0.16
p.adjust
9e−04
6e−04
3e−04
D
forebrain development
telencephalon development
pattern specification process
oligodendrocyte differentiation
adrenal gland development
cellular amino acid metabolic process
protein serine/threonine kinase signaling pathway
regulation of smooth muscle cell differentiation
pallium development
negative regulation of muscle cell differentiation
synapse organization
axonogenesis
synapse assembly
regulation of membrane potential
cognition
learning or memory
regulation of synapse organization
regulation of synapse structure or activity
cell−cell adhesion
cell junction assembly
increase (627)
decrease (1594)
GeneRatio
0.02
0.04
0.06
p.adjust
0.03
0.02
0.01

Figure S3: *Foxg1* haploinsufficiency and shRNA mediated knockdown affect similar and distinct biological functions *in vivo* and *in vitro* and at different developmental stages.

(A) Dotplot of differential GO-term enrichment analysis comparing DEGs in all datasets (***in vitro*** shFoxg1/shLuciferase, $Foxg1^{cre/+}$/WT, ***in vivo*** adult, and E18.5 $Foxg1^{cre/+}$/WT). Total number of genes per group is on the x-axis, dot size displays the gene ratios and color indicates significance level of the enrichment. **(B)** Top ten enriched terms in the fractions of increased and decreased DEGs in the adult $Foxg1^{cre/+}$ hippocampus and DIV11 primary neurons upon FOXG1 KD (B) compared simultaneously (C), and separately (D). **(D)** Shows the top ten enriched GO-terms when the increase/decrease fractions are compared within their own datasets, revealing enriched terms that are not in the top ten when the datasets are compared simultaneously. Threshold for enrichment analyses: $p < 0.05$.

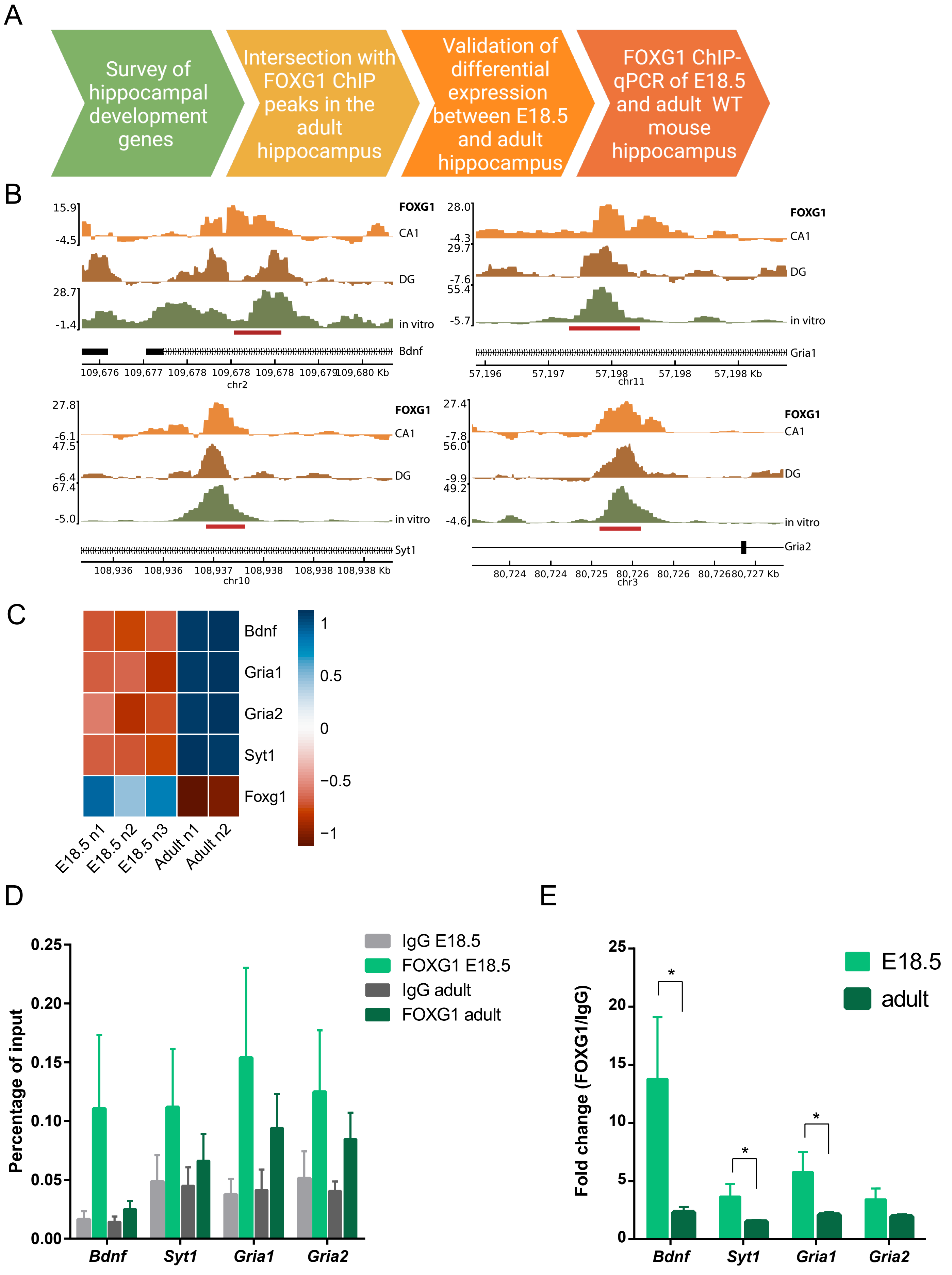
A
Survey of hippocampal development genes
Intersection with FOXG1 ChIP peaks in the adult hippocampus
Validation of differential expression between E18.5 and adult hippocampus
FOXG1 ChIP-qPCR of E18.5 and adult WT mouse hippocampus
B
FOXG1
CA1
DG
in vitro
Bdnf
Gria1
Syt1
Gria2
chr2
chr11
chr10
chr3
C
Bdnf
Gria1
Gria2
Syt1
Foxg1
E18.5 n1
E18.5 n2
E18.5 n3
Adult n1
Adult n2
D
IgG E18.5
FOXG1 E18.5
IgG adult
FOXG1 adult
Percentage of input
Bdnf
Syt1
Gria1
Gria2
E
E18.5
adult
Fold change (FOXG1/IgG)
Bdnf
Syt1
Gria1
Gria2

Figure S4: FOXG1 enrichment on potential hippocampal maturation genes is altered between E18.5 and adult hippocampus.

(A) Pipeline explaining target selection and experimental design. **(B)** Genome browser tracks of CA1 (orange), DG (brown), and *in vitro* (green) FOXG1 peaks at selected target genes: *Bdnf, Syt1, Gria1, and Gria2*. Red line depicts the regions targeted in ChIP-qPCR experiments. **(C)** Heatmap of normalized counts of *Bdnf, Syt1, Gria1, Gria2, and Foxg1* in WT E18.5 and adult hippocampi. Scale in rlog normalized counts (*DESeq2*) (E18.5 n= 3, adult n= 2). **(D)** ChIP-qPCR analysis of FOXG1 levels (% Input) of WT E18.5 and adult hippocampi at the target regions of *Bdnf, Syt1, Gria1, and Gria2* from (B) ($n = 3$). **(E)** ChIP-qPCR results represented as fold change (FOXG1-ChIP/IgG). Data represented as mean ± SEM. Two- tailed Student's t-test: *p< 0.05, **p < 0.01, ***p < 0.001.

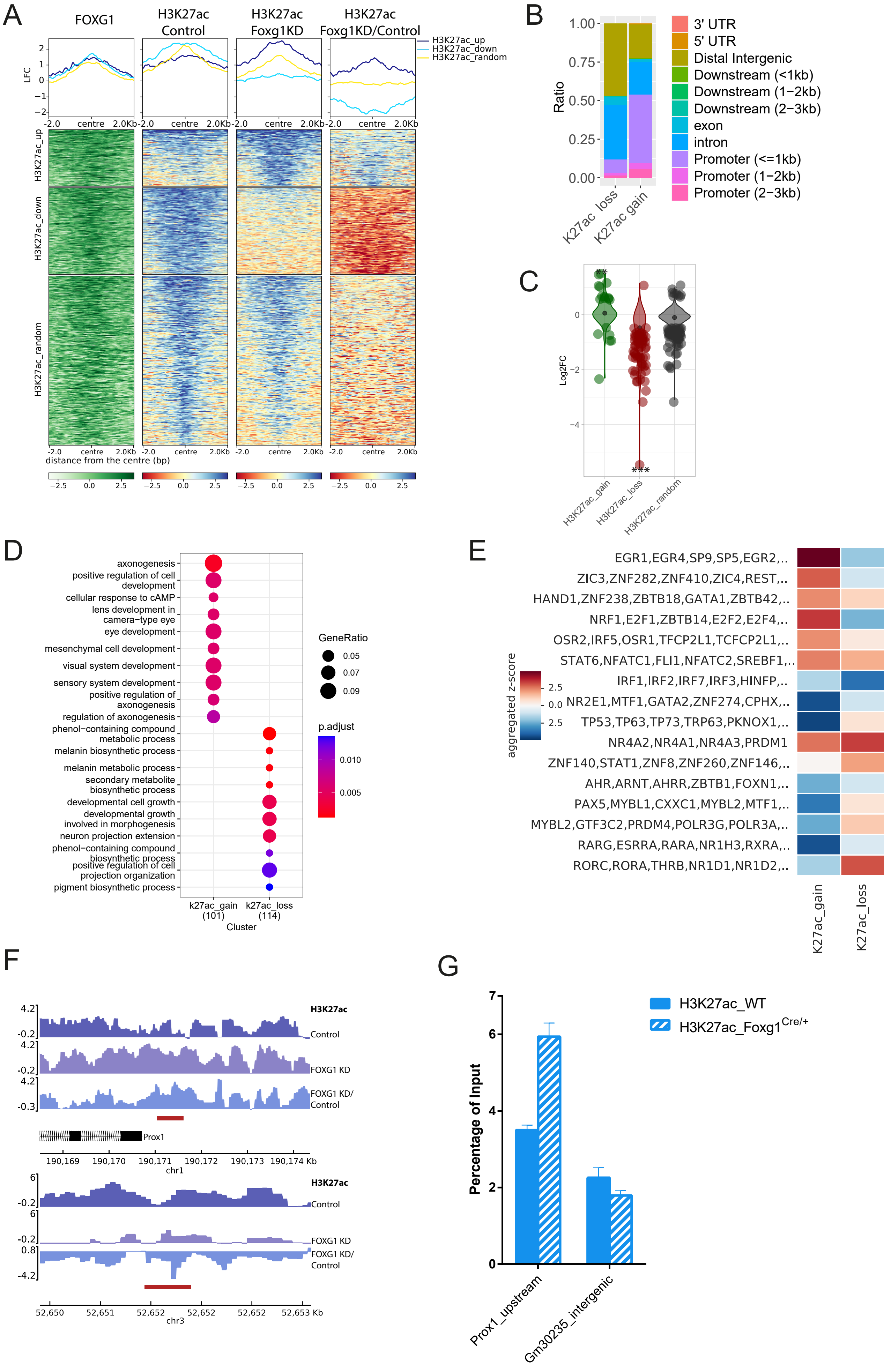
A
FOXG1
H3K27ac Control
H3K27ac Foxg1KD
H3K27ac Foxg1KD/Control
H3K27ac_up
H3K27ac_down
H3K27ac_random
LFC
centre
distance from the centre (bp)
B
Ratio
3' UTR
5' UTR
Distal Intergenic
Downstream (<1kb)
Downstream (1−2kb)
Downstream (2−3kb)
exon
intron
Promoter (<=1kb)
Promoter (1−2kb)
Promoter (2−3kb)
K27ac loss
K27ac gain
C
Log2FC
H3K27ac_gain
H3K27ac_loss
H3K27ac_random
D
axonogenesis
positive regulation of cell development
cellular response to cAMP
lens development in camera−type eye
eye development
mesenchymal cell development
visual system development
sensory system development
positive regulation of axonogenesis
regulation of axonogenesis
phenol−containing compound metabolic process
melanin biosynthetic process
melanin metabolic process
secondary metabolite biosynthetic process
developmental cell growth
developmental growth involved in morphogenesis
neuron projection extension
phenol−containing compound biosynthetic process
positive regulation of cell projection organization
pigment biosynthetic process
GeneRatio
p.adjust
k27ac_gain (101)
k27ac_loss (114)
Cluster
E
EGR1,EGR4,SP9,SP5,EGR2,..
ZIC3,ZNF282,ZNF410,ZIC4,REST,..
HAND1,ZNF238,ZBTB18,GATA1,ZBTB42,..
NRF1,E2F1,ZBTB14,E2F2,E2F4,..
OSR2,IRF5,OSR1,TFCP2L1,TCFCP2L1,..
STAT6,NFATC1,FLI1,NFATC2,SREBF1,..
IRF1,IRF2,IRF7,IRF3,HINFP,..
NR2E1,MTF1,GATA2,ZNF274,CPHX,..
TP53,TP63,TP73,TRP63,PKNOX1,..
NR4A2,NR4A1,NR4A3,PRDM1
ZNF140,STAT1,ZNF8,ZNF260,ZNF146,..
AHR,ARNT,AHRR,ZBTB1,FOXN1,..
PAX5,MYBL1,CXXC1,MYBL2,MTF1,..
MYBL2,GTF3C2,PRDM4,POLR3G,POLR3A,..
RARG,ESRRA,RARA,NR1H3,RXRA,..
RORC,RORA,THRB,NR1D1,NR1D2,..
aggregated z-score
K27ac_gain
K27ac_loss
F
H3K27ac
Control
FOXG1 KD
FOXG1 KD/ Control
Prox1
chr1
chr3
G
H3K27ac_WT
H3K27ac_Foxg1Cre/+
Percentage of Input
Prox1_upstream
Gm30235_intergenic

Figure S5: Reduced FOXG1 levels lead to gain and loss of H3K27ac *in vitro* and *in vivo*.

(A) Heatmap of H3K27ac enrichment at regions retrieved from differential binding analysis of H3K27ac FOXG1 KD/Control (H3K27ac-up, -down, -random). Data is normalized by sequencing depth and input control as log_2 (ChIP/Input) for H3K27ac control and H3K27ac FOXG1 KD data. The difference between FOXG1 KD and control conditions was calculated from RPKM normalized bigwig files as log_2 (FOXG1 KD/Control). The metaprofiles (top) show the average log_2FC (LFC) of each cluster. **(B)** Genomic distribution of regions gaining and losing H3K27ac enrichment displayed as a stacked bar graph. **(C)** Violin plot depicting the distribution of DEGs upon FOXG1 KD at H3K27ac-gain,–loss and -random clusters as shown in A. Y-axis corresponds to log_2FC of gene expression, and x-axis shows the three clusters. The black dot marks the median of log_2FC of DEGs in each cluster. *GeneOverlap Fisher's exact test* *: $p<0.05$, **: $p<0.01$, ***: $p<0.001$. **(D)** Enriched GO-terms for the respective clusters as shown in A. Scales of gene ratios and adjusted p-value are at top-right corner, and total number of genes per cluster are on the x-axis. Threshold for enrichment analysis was adjusted to $p < 0.01$. **(E)** Heatmap showing transcription factor (TF)-binding differential motif analysis according to the clusters of H3K27ac enrichment as shown in A. **(F)** Genome browser tracks of H3K27ac in control, FOXG1 KD and FOXG1 KD/Control conditions at selected target regions for gain (up) and loss (down). Red line depicts the regions targeted in ChIP-qPCR experiments. **(G)** ChIP-qPCR analysis of H3K27ac levels (% Input) of adult WT (solid blue) and $Foxg1^{cre/+}$ hippocampi (grated blue) at the target regions from (A) (n= 2). Data represented as mean ± SEM.

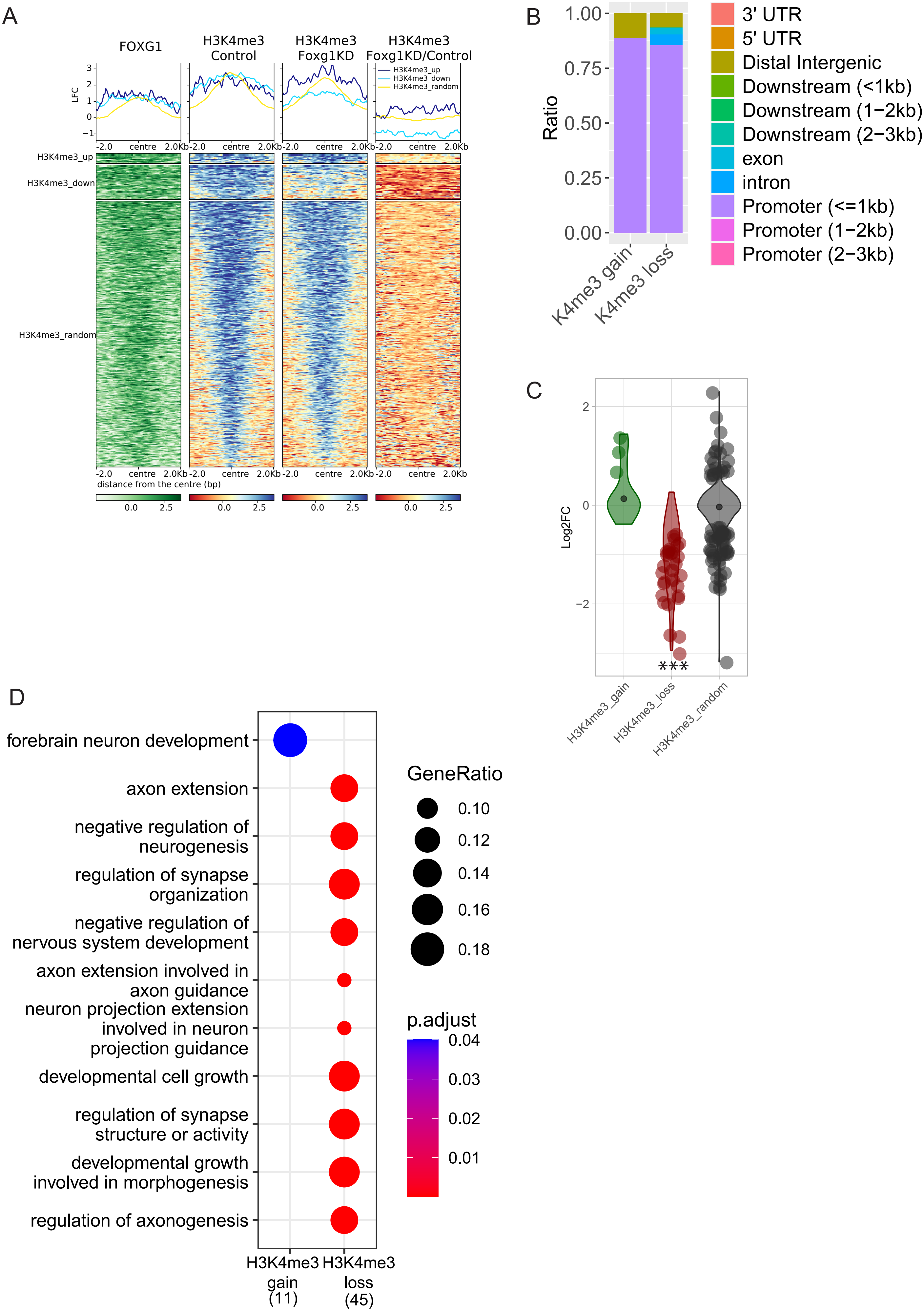
A
FOXG1
H3K4me3 Control
H3K4me3 Foxg1KD
H3K4me3 Foxg1KD/Control
H3K4me3_up
H3K4me3_down
H3K4me3_random
LFC
-2.0 centre 2.0Kb
distance from the centre (bp)
0.0 2.5
B
Ratio
1.00
0.75
0.50
0.25
0.00
K4me3 gain
K4me3 loss
3' UTR
5' UTR
Distal Intergenic
Downstream (<1kb)
Downstream (1−2kb)
Downstream (2−3kb)
exon
intron
Promoter (<=1kb)
Promoter (1−2kb)
Promoter (2−3kb)
C
Log2FC
2
0
−2
***
H3K4me3_gain
H3K4me3_loss
H3K4me3_random
D
forebrain neuron development
axon extension
negative regulation of neurogenesis
regulation of synapse organization
negative regulation of nervous system development
axon extension involved in axon guidance
neuron projection extension involved in neuron projection guidance
developmental cell growth
regulation of synapse structure or activity
developmental growth involved in morphogenesis
regulation of axonogenesis
H3K4me3 gain (11)
H3K4me3 loss (45)
GeneRatio
0.10
0.12
0.14
0.16
0.18
p.adjust
0.04
0.03
0.02
0.01

Figure S6: Reduced FOXG1 levels lead to gain and loss of H3K4me3 *in vitro*.

(A) Heatmap of H3K4me3 enrichment at regions retrieved from differential binding analysis of H3K4me3 FOXG1 KD/Control (H3K4me3-up, -down, -random). Data is normalized by sequencing depth and input control as $\log_2$ (ChIP/Input) for H3K4me3 control and H3K4me3 FOXG1 KD data. The difference between FOXG1 KD and control conditions was calculated from RPKM normalized bigwig files as $\log_2$ (FOXG1 KD/Control). The metaprofiles (top) show the average $\log_2$FC (LFC) of each cluster. **(B)** Genomic distribution of regions gaining and losing H3K4me3 enrichment displayed as a stacked bar graph. **(C)** Violin plot depicting the distribution of DEGs upon FOXG1 KD at H3K4me3-gain,–loss and -random clusters as shown in A. Y-axis corresponds to $\log_2$FC of gene expression, and x-axis shows the three clusters. The black dot marks the median of $\log_2$FC of DEGs in each cluster. *GeneOverlap Fisher's exact test* *: $p<0.05$, **: $p<0.01$, ***: $p<0.001$. **(D)** Enriched GO-terms for the respective clusters as shown in A. Scales of gene ratios and adjusted p-value are at top-right corner, and total number of genes per cluster are on the x-axis. Threshold for enrichment analysis: $p< 0.01$.

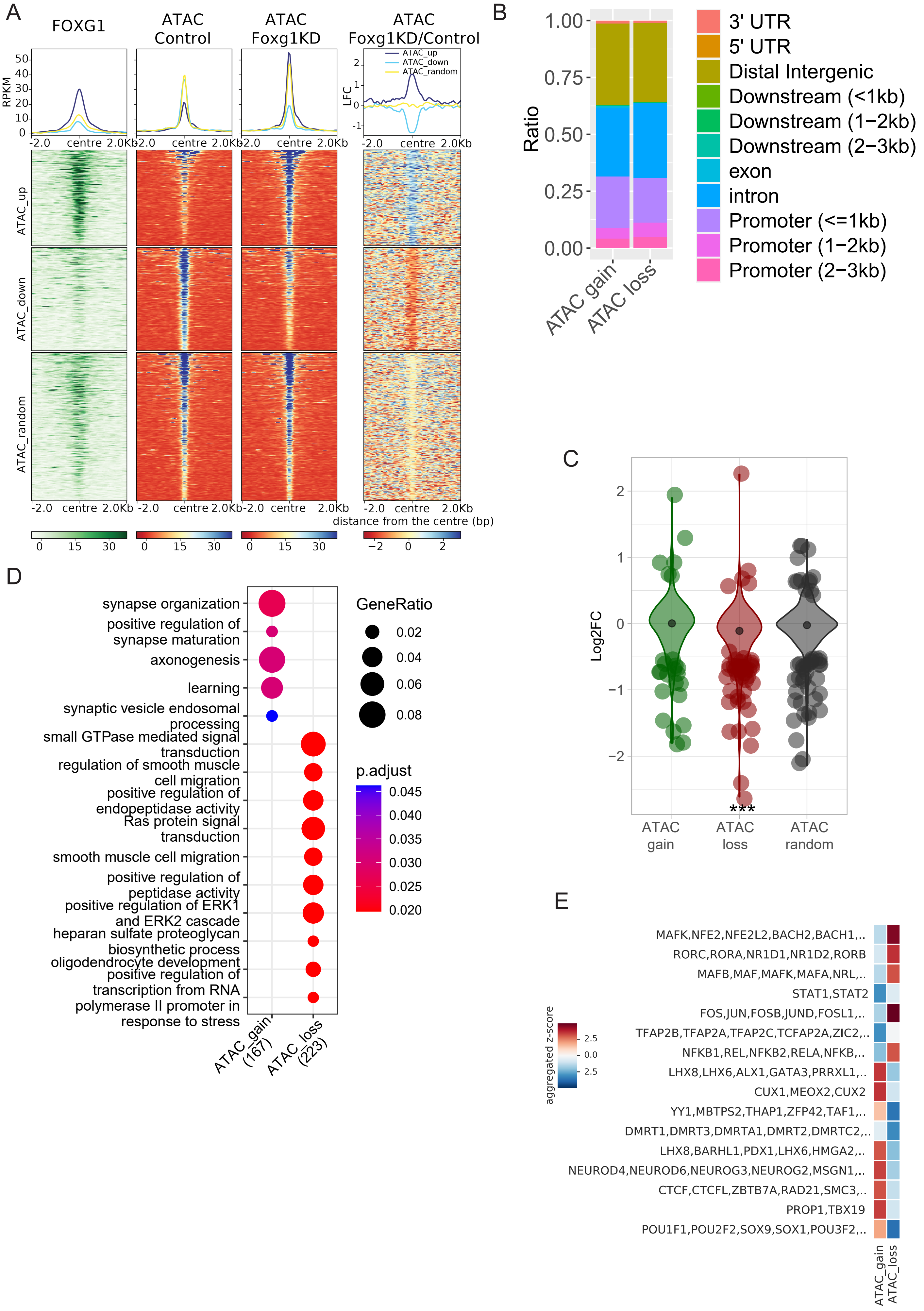

A
FOXG1
ATAC Control
ATAC Foxg1KD
ATAC Foxg1KD/Control
RPKM
LFC
ATAC_up
ATAC_down
ATAC_random
-2.0 centre 2.0Kb
distance from the centre (bp)
B
Ratio
1.00
0.75
0.50
0.25
0.00
ATAC gain
ATAC loss
3' UTR
5' UTR
Distal Intergenic
Downstream (<1kb)
Downstream (1−2kb)
Downstream (2−3kb)
exon
intron
Promoter (<=1kb)
Promoter (1−2kb)
Promoter (2−3kb)
C
Log2FC
ATAC gain
ATAC loss
ATAC random
***
D
synapse organization
positive regulation of synapse maturation
axonogenesis
learning
synaptic vesicle endosomal processing
small GTPase mediated signal transduction
regulation of smooth muscle cell migration
positive regulation of endopeptidase activity
Ras protein signal transduction
smooth muscle cell migration
positive regulation of peptidase activity
positive regulation of ERK1 and ERK2 cascade
heparan sulfate proteoglycan biosynthetic process
oligodendrocyte development
positive regulation of transcription from RNA polymerase II promoter in response to stress
ATAC_gain (167)
ATAC_loss (223)
GeneRatio
0.02
0.04
0.06
0.08
p.adjust
0.045
0.040
0.035
0.030
0.025
0.020
E
aggregated z-score
MAFK,NFE2,NFE2L2,BACH2,BACH1,..
RORC,RORA,NR1D1,NR1D2,RORB
MAFB,MAF,MAFK,MAFA,NRL,..
STAT1,STAT2
FOS,JUN,FOSB,JUND,FOSL1,..
TFAP2B,TFAP2A,TFAP2C,TCFAP2A,ZIC2,..
NFKB1,REL,NFKB2,RELA,NFKB,..
LHX8,LHX6,ALX1,GATA3,PRRXL1,..
CUX1,MEOX2,CUX2
YY1,MBTPS2,THAP1,ZFP42,TAF1,..
DMRT1,DMRT3,DMRTA1,DMRT2,DMRTC2,..
LHX8,BARHL1,PDX1,LHX6,HMGA2,..
NEUROD4,NEUROD6,NEUROG3,NEUROG2,MSGN1,..
CTCF,CTCFL,ZBTB7A,RAD21,SMC3,..
PROP1,TBX19
POU1F1,POU2F2,SOX9,SOX1,POU3F2,..
ATAC_gain
ATAC_loss

Figure S7: Reduced FOXG1 levels lead to gain and loss of chromatin accessibility *in vitro*.

(A) Heatmap of chromatin accessibility enrichment at regions retrieved from differential binding analysis of ATAC FOXG1 KD/Control (ATAC-up, -down, -random). Data is normalized by sequencing depth as RPKM for ATAC control and ATAC FOXG1 KD data. The difference between FOXG1 KD and control conditions was calculated from RPKM normalized bigwig files as $\log_2$(FOXG1 KD/Control). The metaprofiles (top) show the average $\log_2$FC (LFC) of each cluster. **(B)** Genomic distribution of regions gaining and losing accessibility displayed as a stacked bar graph. **(C)** Violin plot depicting the distribution of DEGs upon FOXG1 KD at ATAC-gain,–loss and -random clusters as shown in A. Y-axis corresponds to $\log_2$FC of gene expression, and x-axis shows the three clusters. The black dot marks the median of $\log_2$FC of DEGs in each cluster. *GeneOverlap Fisher's exact test* *: $p<0.05$, **: $p<0.01$, ***: $p<0.001$. **(D)** Enriched GO-terms for the respective clusters as shown in A. Scales of gene ratios and adjusted p-value are at top-right corner, and total number of genes per cluster are on the x-axis. Threshold for enrichment analysis: $p< 0.01$. **(E)** Heatmap showing transcription factor (TF)-binding differential motif analysis according to the clusters of chromatin accessibility as shown in A.

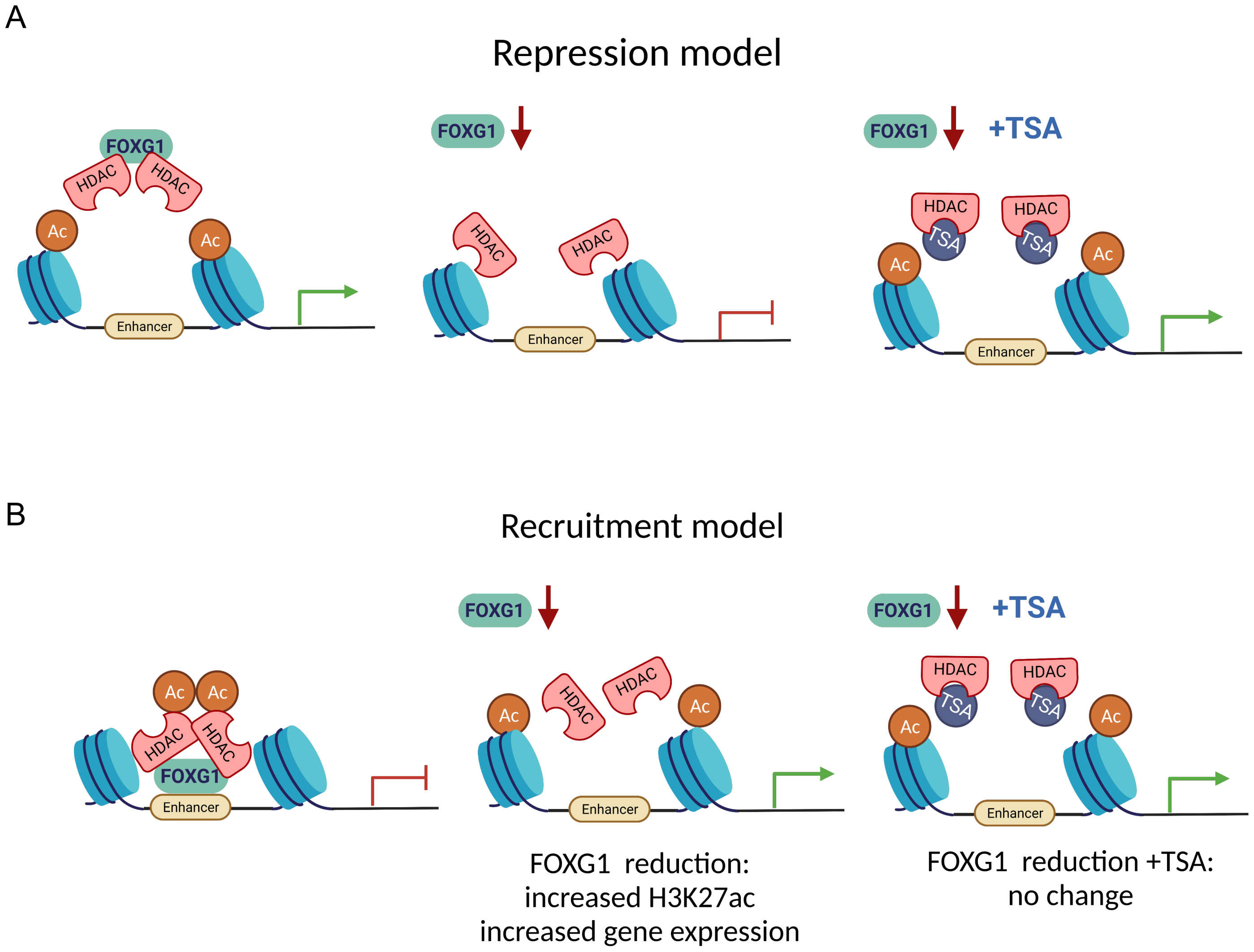
A
Repression model
FOXG1
HDAC
HDAC
Ac
Ac
Enhancer
FOXG1
HDAC
HDAC
Enhancer
FOXG1
+TSA
HDAC
HDAC
TSA
TSA
Ac
Ac
Enhancer
B
Recruitment model
Ac
Ac
HDAC
HDAC
FOXG1
Enhancer
FOXG1
HDAC
HDAC
Ac
Ac
Enhancer
FOXG1
+TSA
HDAC
HDAC
TSA
TSA
Ac
Ac
Enhancer
FOXG1 reduction:
increased H3K27ac
increased gene expression
FOXG1 reduction +TSA:
no change

Figure S8: Repression and recruitment models of HDAC-FOXG1 interaction.

Graphical summary of the repression **(A)** and recruitment **(B)** models**. (A)** The repression model is independent of FOXG1-binding to the chromatin and predicts that reduced levels of FOXG1 lead to reduced levels of H3K27ac and concomitant transcriptional decrease. Upon HDAC inhibition with TSA, we expect transcriptional increase of genes regulated through the repression model. **(B)** The recruitment model predicts that reduced levels of FOXG1 correlate with increased H3K27ac levels and concomitantly with increased transcription at FOXG1-binding. TSA treatment would not alter transcription upon reduced FOXG1 levels, as inhibition of the HDACs does not occur bound to the chromatin.

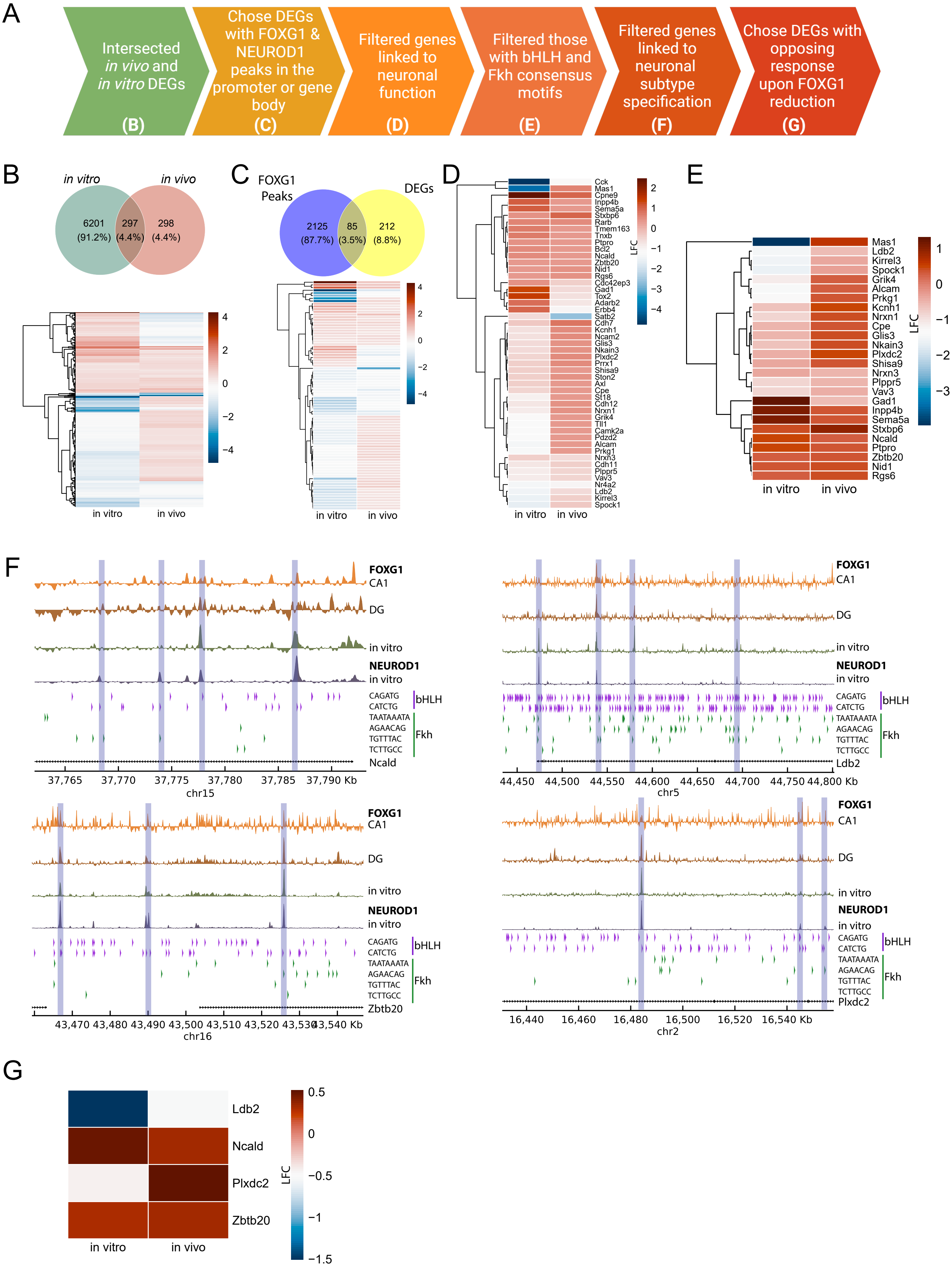
A
Intersected in vivo and in vitro DEGs (B)
Chose DEGs with FOXG1 & NEUROD1 peaks in the promoter or gene body (C)
Filtered genes linked to neuronal function (D)
Filtered those with bHLH and Fkh consensus motifs (E)
Filtered genes linked to neuronal subtype specification (F)
Chose DEGs with opposing response upon FOXG1 reduction (G)
B
in vitro
in vivo
6201 (91.2%)
297 (4.4%)
298 (4.4%)
in vitro
in vivo
C
FOXG1 Peaks
DEGs
2125 (87.7%)
85 (3.5%)
212 (8.8%)
in vitro
in vivo
D
Cck
Mas1
Cpne9
Inpp4b
Sema5a
Stxbp6
Rarb
Tmem163
Tnxb
Ptpro
Bcl2
Ncald
Zbtb20
Nid1
Rgs6
Cdc42ep3
Gad1
Tox2
Adarb2
Erbb4
Satb2
Cdh7
Kcnh1
Ncam2
Glis3
Nkain3
Plxdc2
Prrx1
Shisa9
Ston2
Axl
Cpe
St18
Cdh12
Nrxn1
Grik4
Tll1
Camk2a
Pdzd2
Alcam
Prkg1
Nrxn3
Cdh11
Plppr5
Vav3
Nr4a2
Ldb2
Kirrel3
Spock1
LFC
in vitro
in vivo
E
Mas1
Ldb2
Kirrel3
Spock1
Grik4
Alcam
Prkg1
Kcnh1
Nrxn1
Cpe
Glis3
Nkain3
Plxdc2
Shisa9
Nrxn3
Plppr5
Vav3
Gad1
Inpp4b
Sema5a
Stxbp6
Ncald
Ptpro
Zbtb20
Nid1
Rgs6
LFC
in vitro
in vivo
F
FOXG1
CA1
DG
in vitro
NEUROD1
in vitro
CAGATG
CATCTG
bHLH
TAATAAATA
AGAACAG
TGTTTAC
TCTTGCC
Fkh
Ncald
37,765 37,770 37,775 37,780 37,785 37,790 Kb
chr15
Ldb2
44,450 44,500 44,550 44,600 44,650 44,700 44,750 44,800 Kb
chr5
Zbtb20
43,470 43,480 43,490 43,500 43,510 43,520 43,530 43,540 Kb
chr16
Plxdc2
16,440 16,460 16,480 16,500 16,520 16,540 Kb
chr2
G
Ldb2
Ncald
Plxdc2
Zbtb20
in vitro
in vivo
LFC
0.5
0
−0.5
−1
−1.5

Figure S9: Determination of *Ldb2* and *Ncald* as FOXG1 regulatory targets.

(A) Pipeline of analyses used for the determination of potential direct regulatory targets of FOXG1, showing each panel relating to the steps. **(B)** Intersection of DEGs (LFC cut-off ±0.3) from *in vivo* and *in vitro* datasets yielded 297 genes shown in the Venn diagram (top). Comparison of expression levels (LFC) between *in vivo* and *in vitro* datasets plotted in a heatmap (bottom). **(C)** DEGs with FOXG1 and NEUROD1 in the promoter or gene body were chosen, and expression levels were depicted in the heatmap. Scale same as in B. These DEGs were filtered by their link to neuronal function (heatmap) **(D)** and occurrence of Fkh and bHLH motifs with overlapping FOXG1 and NEUROD1 peaks (heatmap) **(E)**. The FOXG1 and NEUROD1 occupancy was plotted for genes with specific link to neuronal subtype specification and adjacent motifs of bHLH and Fkh motifs within **maximum 200 bp** of each other (*Ncald, Ldb2, Plxdc2* and *Zbtb20*) **(F)**. **(G)** *Ldb2* was the only gene that was downregulated upon reduced levels of FOXG1 (activator function of FOXG1), and *Ncald* was chosen for the repressor function of FOXG1 for the luciferase assays in **Figure 5**.

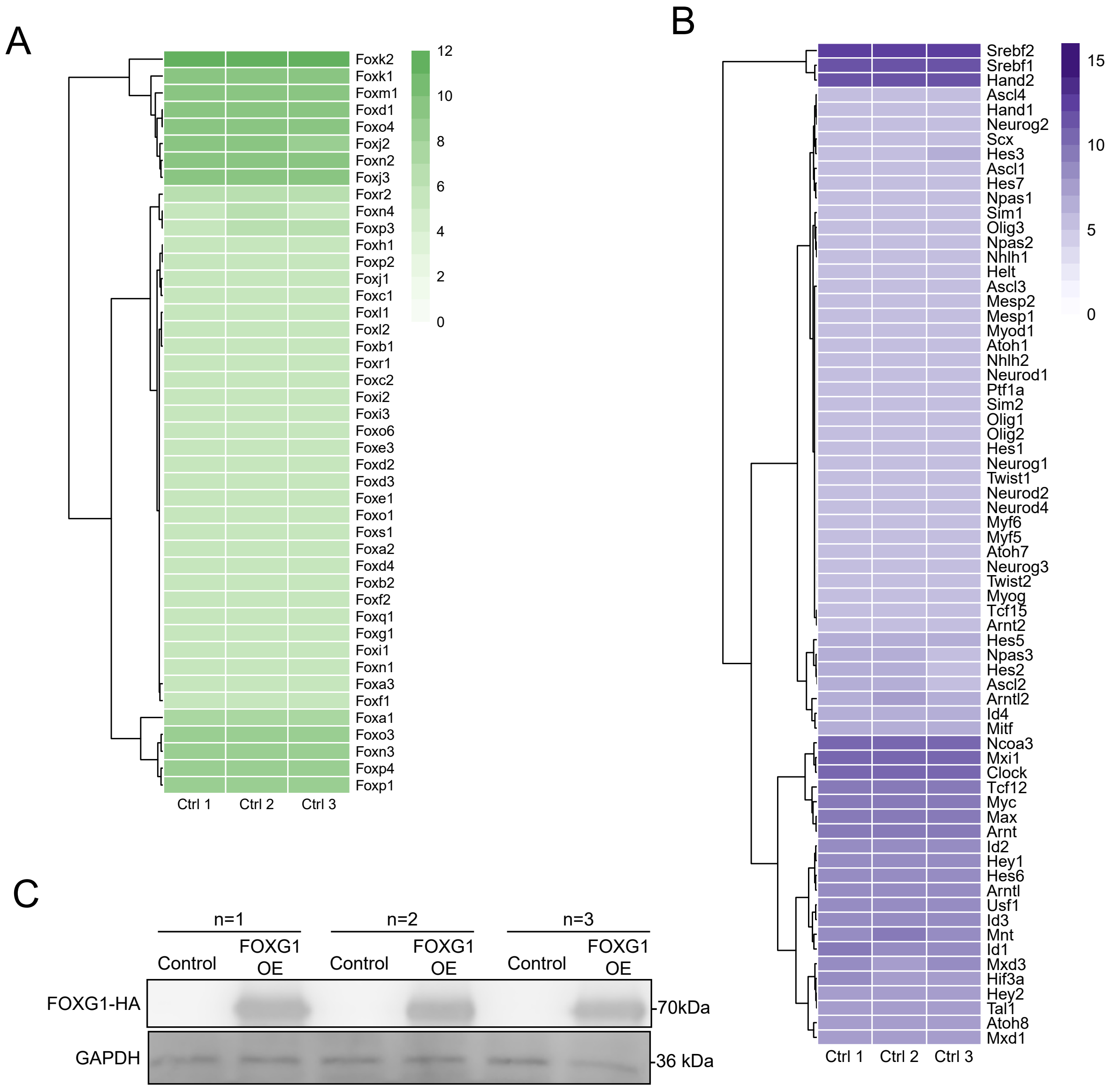

A
Foxk2
Foxk1
Foxm1
Foxd1
Foxo4
Foxj2
Foxn2
Foxj3
Foxr2
Foxn4
Foxp3
Foxh1
Foxp2
Foxj1
Foxc1
Foxl1
Foxl2
Foxb1
Foxr1
Foxc2
Foxi2
Foxi3
Foxo6
Foxe3
Foxd2
Foxd3
Foxe1
Foxo1
Foxs1
Foxa2
Foxd4
Foxb2
Foxf2
Foxq1
Foxg1
Foxi1
Foxn1
Foxa3
Foxf1
Foxa1
Foxo3
Foxn3
Foxp4
Foxp1
Ctrl 1
Ctrl 2
Ctrl 3
12
10
8
6
4
2
0
B
Srebf2
Srebf1
Hand2
Ascl4
Hand1
Neurog2
Scx
Hes3
Ascl1
Hes7
Npas1
Sim1
Olig3
Npas2
Nhlh1
Helt
Ascl3
Mesp2
Mesp1
Myod1
Atoh1
Nhlh2
Neurod1
Ptf1a
Sim2
Olig1
Olig2
Hes1
Neurog1
Twist1
Neurod2
Neurod4
Myf6
Myf5
Atoh7
Neurog3
Twist2
Myog
Tcf15
Arnt2
Hes5
Npas3
Hes2
Ascl2
Arntl2
Id4
Mitf
Ncoa3
Mxi1
Clock
Tcf12
Myc
Max
Arnt
Id2
Hey1
Hes6
Arntl
Usf1
Id3
Mnt
Id1
Mxd3
Hif3a
Hey2
Tal1
Atoh8
Mxd1
Ctrl 1
Ctrl 2
Ctrl 3
15
10
5
0
C
n=1
n=2
n=3
Control
FOXG1 OE
Control
FOXG1 OE
Control
FOXG1 OE
FOXG1-HA
GAPDH
70kDa
36 kDa

Figure S10: Fkh and bHLH transcription factors are expressed at moderate levels in N2A cells.

Heatmap of normalized counts of **(A)** Fkh and **(B)** bHLH TFs in N2A cells (n=3). **(C)** Immunoblot confirming the overexpression of FOXG1 using FOXG1 in N2A cells (n=3).

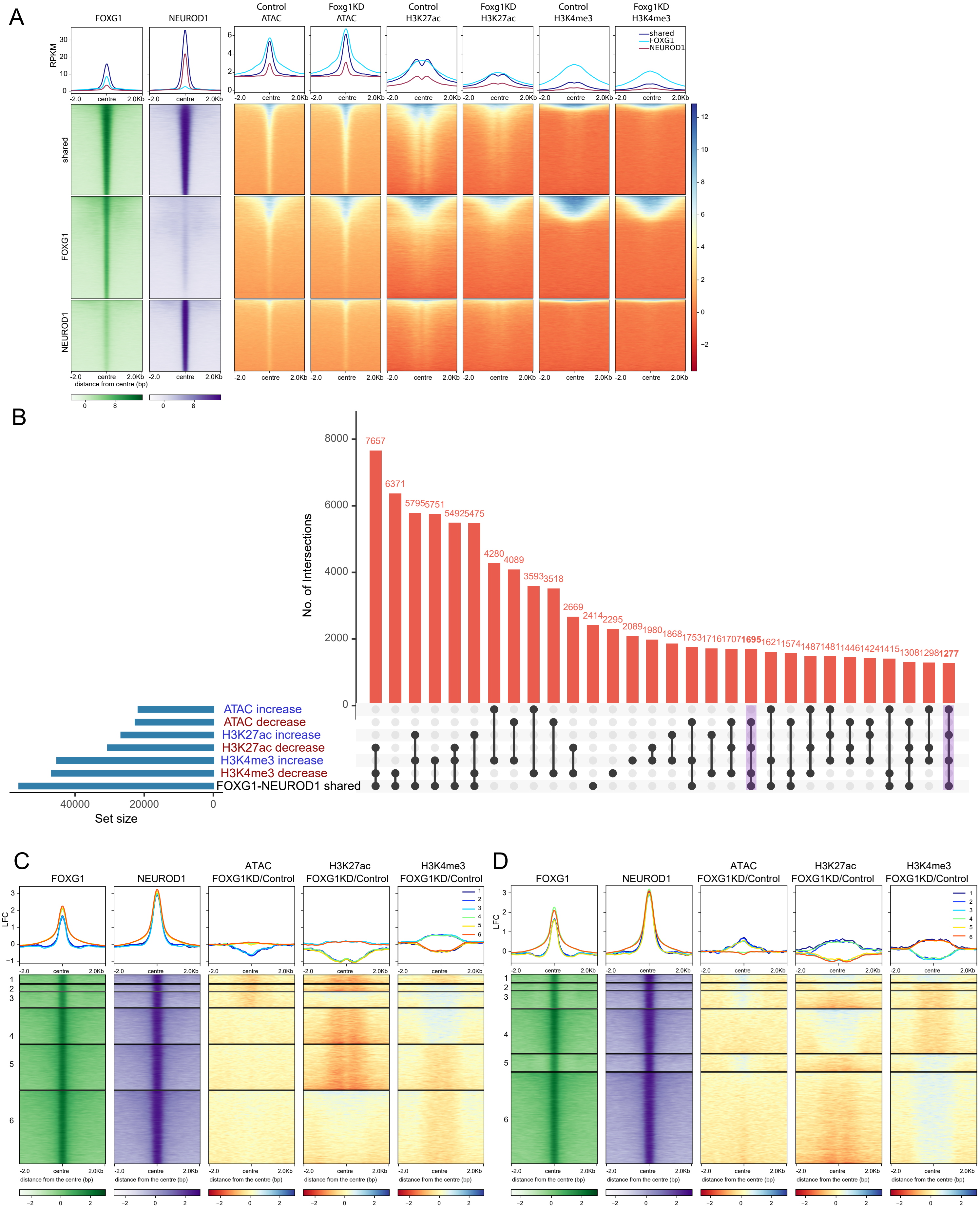
A
FOXG1
NEUROD1
Control ATAC
Foxg1KD ATAC
Control H3K27ac
Foxg1KD H3K27ac
Control H3K4me3
Foxg1KD H3K4me3
shared
FOXG1
NEUROD1
RPKM
-2.0
centre
2.0Kb
distance from centre (bp)
B
No. of Intersections
7657
6371
5795
5751
5492
5475
4280
4089
3593
3518
2669
2414
2295
2089
1980
1868
1753
1716
1707
1695
1621
1574
1487
1481
1446
1424
1415
1308
1298
1277
ATAC increase
ATAC decrease
H3K27ac increase
H3K27ac decrease
H3K4me3 increase
H3K4me3 decrease
FOXG1-NEUROD1 shared
Set size
C
D
FOXG1
NEUROD1
ATAC FOXG1KD/Control
H3K27ac FOXG1KD/Control
H3K4me3 FOXG1KD/Control
LFC
distance from the centre (bp)

Figure S11: Reduced FOXG1 levels affect the epigenetic landscape in FOXG1-NEUROD1 shared binding regions.

(A) Heatmap showing chromatin accessibility, H3K27ac, H3K4me3 enrichment at FOXG1 (green) and NEUROD1 (purple) binding sites clustered into shared and unique (FOXG1_unique, NEUROD1_unique) regions in control and FOXG1 KD conditions. **(B)** Upset plots depicting the multi-intersection between FOXG1_unique (top), NEUROD1_unique (middle), and shared (bottom) binding sites with dynamic clusters of H3K27ac, H3K4me3, and ATAC from Fig. 3. **(C, D)** Heatmaps showing chromatin accessibility, H3K27ac, H3K4me3 enrichment differences between FOXG1 KD/Control conditions (log2 (FOXG1 KD/Control)) at sub-clusters of shared FOXG1 and NEUROD1 binding sites. The clusters were retrieved by multi-intersecting the FOXG1-NEUROD1 shared regions with dynamic decrease (C) or increase (D) clusters of H3K27ac, H3K4me3 and ATAC from Fig. 3. Data normalization and metaprofiles (top) as in S5F.

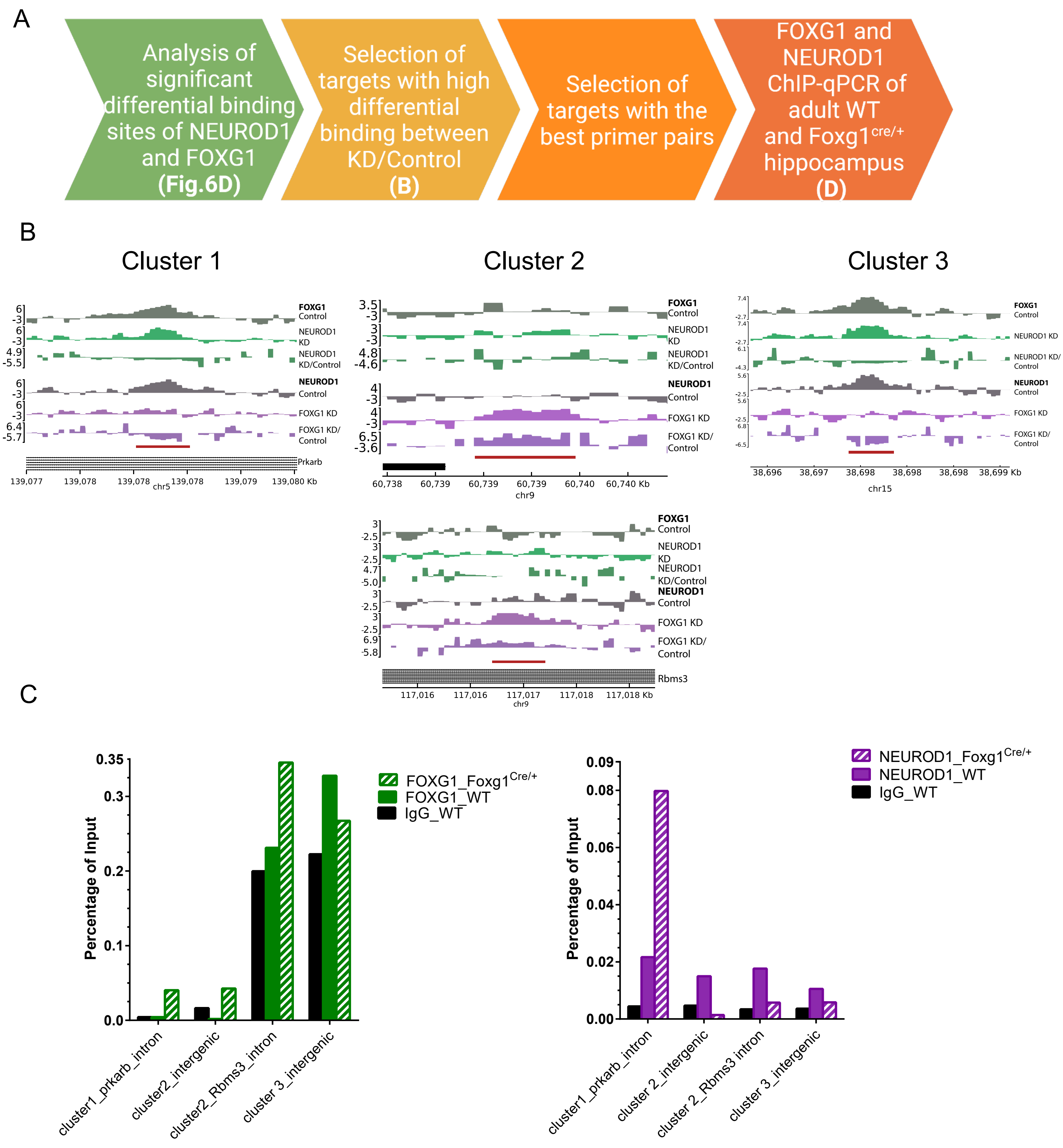
A
Analysis of significant differential binding sites of NEUROD1 and FOXG1 (Fig.6D)
Selection of targets with high differential binding between KD/Control (B)
Selection of targets with the best primer pairs
FOXG1 and NEUROD1 ChIP-qPCR of adult WT and Foxg1cre/+ hippocampus (D)
B
Cluster 1
Cluster 2
Cluster 3
FOXG1 Control
NEUROD1 KD
NEUROD1 KD/Control
NEUROD1 Control
FOXG1 KD
FOXG1 KD/Control
Prkarb
chr5
chr9
chr15
Rbms3
C
Percentage of Input
FOXG1_Foxg1Cre/+
FOXG1_WT
IgG_WT
cluster1_prkarb_intron
cluster2_intergenic
cluster2_Rbms3_intron
cluster 3_intergenic
NEUROD1_Foxg1Cre/+
NEUROD1_WT
IgG_WT
cluster 2_intergenic
cluster 2_Rbms3 intron

Figure S12: NEUROD1 and FOXG1 bind the chromatin cooperatively and competitively *in vivo.*

(A) Pipeline explaining target selection and experimental design of FOXG1-NEUROD1 ChIP-qPCR. **(B)** Genome browser tracks of FOXG1 and NEUROD1 peaks at selected target regions for cluster 1 (left), cluster 2 (middle), and cluster 3 (left). Track labels are on the right of plots. Red line depicts the regions targeted in ChIP-qPCR experiments. **(C)** FOXG1 (left) and NEUROD1 (right) ChIP-qPCR analysis of FOXG1 levels (% Input) of adult WT and Foxg1$^{cre/+}$ hippocampi at the target regions from (B) (n= 1). Data represented as mean of technical replicates.

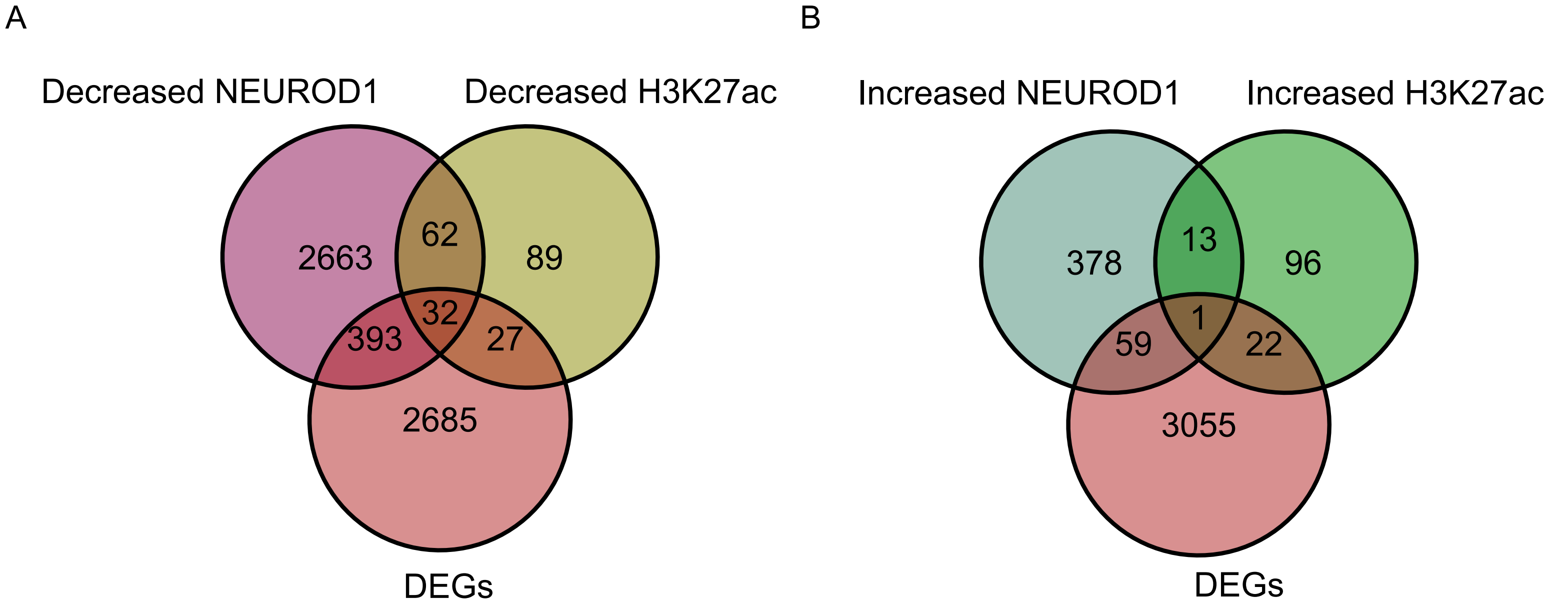
A
Decreased NEUROD1
Decreased H3K27ac
2663
62
89
32
393
27
2685
DEGs
B
Increased NEUROD1
Increased H3K27ac
378
13
96
1
59
22
3055
DEGs

Figure S13: Intersection of differential NEUROD1 and H3K27ac binding, and differential gene expression upon FOXG1 KD.
(A) Venn diagram of decreased NEUROD1 (pink) and H3K27ac (yellow), and differential gene expression (red) upon FOXG1 KD. **(B)** Venn diagram of increased NEUROD1 (turquoise) and H3K27ac (green), and differential gene expression (red) upon FOXG1 KD.

References to supplementary data

1. S. C. Weise, *et al.*, FOXG1 Regulates PRKAR2B Transcriptionally and Posttranscriptionally via miR200 in the Adult Hippocampus. *Mol Neurobiol* **56**, 5188–5201 (2019).

2. L. Arrigoni, *et al.*, RELACS nuclei barcoding enables high-throughput ChIP-seq. *Commun Biol* **1**, 214 (2018).

3. J. Seoane, H.-V. Le, L. Shen, S. A. Anderson, J. Massagué, Integration of Smad and Forkhead Pathways in the Control of Neuroepithelial and Glioblastoma Cell Proliferation. *Cell* **117**, 211–223 (2004).

4. C. Liu, *et al.*, Dysregulation of PINCH signaling in mesial temporal epilepsy. *J Clin Neurosci* **36**, 43–52 (2017).

5. J. D. Buenrostro, B. Wu, H. Y. Chang, W. J. Greenleaf, ATAC-seq: A Method for Assaying Chromatin Accessibility Genome-Wide. *Curr Protoc Mol Biol* **109**, 21.29.1-21.29.9 (2015).

6. L. Shen, I. S. of M. at M. S. <shenli sam at gmail.com>, *GeneOverlap: Test and visualize gene overlaps* (Bioconductor version: Release (3.13), 2021) https:/doi.org/10.18129/B9.bioc.GeneOverlap (October 17, 2021).

7. A. R. Quinlan, I. M. Hall, BEDTools: a flexible suite of utilities for comparing genomic features. *Bioinformatics* **26**, 841–842 (2010).

8. E. S. Lein, *et al.*, Genome-wide atlas of gene expression in the adult mouse brain. *Nature* **445**, 168–176 (2007).

9. V. Bhardwaj, *et al.*, snakePipes: facilitating flexible, scalable and integrative epigenomic analysis. *Bioinformatics* **35**, 4757–4759 (2019).

10. F. Ferrari, *et al.*, DOT1L-mediated murine neuronal differentiation associates with H3K79me2 accumulation and preserves SOX2-enhancer accessibility. *Nat Commun* **11**, 5200 (2020).

11. M. I. Love, W. Huber, S. Anders, Moderated estimation of fold change and dispersion for RNA-seq data with DESeq2. *Genome Biology* **15**, 550 (2014).

12. Y. Liao, G. K. Smyth, W. Shi, featureCounts: an efficient general purpose program for assigning sequence reads to genomic features. *Bioinformatics* **30**, 923–930 (2014).

13. F. Ramírez, *et al.*, deepTools2: a next generation web server for deep-sequencing data analysis. *Nucleic Acids Res* **44**, W160-165 (2016).

14. A. T. L. Lun, G. K. Smyth, De novo detection of differentially bound regions for ChIP-seq data using peaks and windows: controlling error rates correctly. *Nucleic Acids Res* **42**, e95 (2014).

15. E. Afgan, *et al.*, The Galaxy platform for accessible, reproducible and collaborative biomedical analyses: 2016 update. *Nucleic Acids Res* **44**, W3–W10 (2016).

16. R. Stark, G. Brown, *DiffBind: Differential Binding Analysis of ChIP-Seq Peak Data* (Bioconductor version: Release (3.13), 2021) https:/doi.org/10.18129/B9.bioc.DiffBind (October 17, 2021).

17. G. Yu, L.-G. Wang, Q.-Y. He, ChIPseeker: an R/Bioconductor package for ChIP peak annotation, comparison and visualization. *Bioinformatics* **31**, 2382–2383 (2015).

18. A. Khan, A. Mathelier, Intervene: a tool for intersection and visualization of multiple gene or genomic region sets. *BMC Bioinformatics* **18**, 287 (2017).

19. S. J. van Heeringen, G. J. C. Veenstra, GimmeMotifs: a de novo motif prediction pipeline for ChIP-sequencing experiments. *Bioinformatics* **27**, 270–271 (2011).

20. T. Wu, *et al.*, clusterProfiler 4.0: A universal enrichment tool for interpreting omics data. *The Innovation* **2**, 100141 (2021).

21. CRAN. R Kolde, pheatmap: Pretty Heatmaps. R package version 1.0. 12 (2019).

22. B. K. Lewis M Rana S., EnhancedVolcano: Publication-ready volcano plots with enhanced colouring and labeling. R package version 1.14.0, (2022).

23. , ggplot2 | SpringerLink (June 30, 2022).

24. Linlin Yan (2021)., ggvenn: Draw Venn Diagram by “ggplot2”. R package version 0.1.9.

25. Hanbo Chen, VennDiagram: Generate High-Resolution Venn and Euler Plots. R package version 1.7.3. (2022).

26. M. S. Cembrowski, L. Wang, K. Sugino, B. C. Shields, N. Spruston, Hipposeq: a comprehensive RNA-seq database of gene expression in hippocampal principal neurons. *eLife* **5**, e14997 (2016).

27. D. Szklarczyk, *et al.*, The STRING database in 2021: customizable protein-protein networks, and functional characterization of user-uploaded gene/measurement sets. *Nucleic Acids Res* **49**, D605–D612 (2021).
